# The co-localizing Zorya II, Druantia III, and ARMADA II defense systems on O-island 172 confer synergistic anti-phage defense in enterohemorrhagic *Escherichia coli*

**DOI:** 10.1128/mbio.00943-26

**Published:** 2026-06-22

**Authors:** Huihui Li, Vu Van Loi, Costanza Borelli, Stephanie Himpich, Michaela Projahn, Lena M. Grass, Thi Phuong Thao Nguyen, Markus C. Wahl, Haike Antelmann

**Affiliations:** 1 Freie Universität Berlin, Institute of Biology-Microbiology 9166, Berlin, Germany; 2 Laboratory of Structural Biochemistry, Freie Universität Berlin 9166, Berlin, Germany; 3 National Reference Laboratory for Escherichia coli Including VTEC, German Federal Institute for Risk Assessment 27652, Berlin, Germany; 4 Helmholtz-Zentrum Berlin für Materialien und Energie, Macromolecular Crystallography 28340, Berlin, Germany; Ludwig-Maximilians-Universitat Munchen, Munich, Germany

**Keywords:** enterohemorrhagic *Escherichia coli* O157:H7, EDL933, OI-172, anti-phage defense systems, Zorya II, Druantia III, ARMADA II, synergy

## Abstract

In this study, we found that the enterohemorrhagic *Escherichia coli* (EHEC) strain EDL933 Δ*stx1/2* is highly resistant to many phages. The three phage defense systems Zorya II, Druantia III, and DISARM-related Antiviral Defense Array (ARMADA II) are encoded on O-island 172 (OI-172) in strain EDL933. Sequence comparison revealed that the three phage defense systems co-occur in 426 *E. coli* strains, including 277 O157:H7 isolates and 117 diverse non-pathogenic strains. Efficiency of plating (EOP) assays was used to explore the phage spectra and synergy of the three defense systems, and their contribution to the phage resistance of the host strain EDL933 Δ*stx1/2*. Using Δ*zorABE*, Δ*druHE*, and Δ*armABCD* single system deletion mutants in EDL933 Δ*stx1/2*, we showed that Druantia III and ARMADA II significantly protect to different extents against a similar spectrum of phages, including the *Drexlerviridae*, *Queuovirinae*, *Demerecviridae*, *Vequintavirinae*, *Stephanstirmvirinae*, and *Autographivirales*, whereas Zorya II only provided protection against *Autographivirales*. Further EOP and growth experiments indicated significantly synergistic and additive interactions of Druantia III and ARMADA II against a broad phage spectrum. The defense pair Druantia III and Zorya II was found to interact additively against a few phages. Altogether, our results revealed that Druantia III and ARMADA II are responsible for most of the phage resistance of EDL933 Δ*stx1/2*, whereas Zorya II provides additional immunity against *Autographivirales*. Future studies are underway to elucidate the molecular basis of the synergistic interactions between the Druantia III and ARMADA II defense systems.

**IMPORTANCE:**

Shiga toxin-producing *Escherichia coli* strains cause life-threatening diseases, such as hemorrhagic colitis and the hemolytic uremic syndrome. Currently, enterohemorrhagic *E. coli* (EHEC) infections can only be treated symptomatically, since antibiotics are not recommended due to induction of the Shiga toxin. While phage therapy could offer a treatment option, we found that the EHEC strain EDL933 Δ*stx1/2* is highly resistant to many phages. Efficiency of plating (EOP) assays of defense mutants in the host strain revealed that the co-occurring defense systems Zorya II, Druantia III, and DISARM-related Antiviral Defense Array (ARMADA II) on O-island 172 (OI-172) confer most of the phage resistance in EHEC. Moreover, Druantia III and ARMADA II were shown to interact additively and synergistically in the anti-phage defense in EDL933 Δ*stx1/2*, providing a more robust immunity than each system alone. These results support the idea that the discovery of inhibitors or anti-defense proteins against the Druantia III and ARMADA II systems could be combined with phage therapies to efficiently eradicate EHEC.

## INTRODUCTION

Enterohemorrhagic *Escherichia coli* (EHEC) O157:H7 is a foodborne and zoonotic pathogen that is transmitted to humans through contaminated milk, meat, or plant-based food and may cause gastrointestinal infections, ranging from diarrhea to life-threatening hemorrhagic colitis and the hemolytic uremic syndrome (HUS) (1–5). EHEC strains are a subgroup of Shiga toxin-producing *E. coli* (STEC), which cause severe outbreaks worldwide at a low infectious dose (<100 cfu) with many hospitalizations and a death rate of 3%–5% (3, 6–9). The Robert Koch Institute in Berlin has recently reported a larger EHEC/HUS outbreak in Germany (10).

However, treatment options for EHEC infections with antibiotics are limited, since many antibiotics induce the Shiga toxin, which enhances the risk for HUS development (11, 12). In addition, current therapies with coliphages against O157:H7 infections only led to modest bacterial killing, indicating that more specific phages against EHEC have to be isolated and alternative treatment strategies developed (13). Thus far, >130 anti-EHEC phages have been isolated from fecal samples of infected human patients and cattle, contaminated wastewater, food, and sewage (14, 15).

The constant arms race between bacteria and their phages has driven the evolution of hundreds of anti-phage defense systems in bacteria that provide innate and adaptive immunity toward phage infection (16–20). The mechanisms of prokaryotic phage defense systems are remarkably complex, including sensing of phage DNA or proteins, signaling through production of small molecules, depletion of nucleotides, chemical defense, or systems sensing the integrity of the host cell upon phage infection (16–20). The most abundant anti-phage defenses in bacteria are nuclease-based systems involved in degradation of phage nucleic acids, including restriction/modification (RM) systems, the Defense Island System Associated with the RM (DISARM), CRISPR-Cas, Zorya, and Druantia defense systems (16–20). In the type I–III RM systems, methyltransferases (MTases) methylate adenine or cytosine bases in the host genome to discriminate non-methylated phage DNA, which is degraded by cognate restriction endonucleases at the unmodified restriction sites (16–18).

In the DISARM system, unmodified DNA substrates with single-stranded overhangs are recognized by the DrmAB helicase complex (21). The Druantia anti-phage defense systems were classified into Druantia types I, II, and III (22). The Druantia III system encompasses the unknown function protein DruH (Z5897) and the very large ATP-dependent DExH box helicase/nuclease DruE (Z5898) containing a DUF1998 and two PLD-like domains. The DUF1998 domain has previously been shown to constitute a Zn^2+^-binding element required for helicase activity in the *Bacillus subtilis* mitomycin repair factor A by enabling a skipping rope-like translocation mechanism (23). Based on the domain architecture, DruE resembles a fusion of the DISARM I components DrmA (helicase), DrmB (DUF1998 protein), and a duplicated DrmC (PLD-containing protein). Druantia III is widely distributed in pathogenic proteobacteria, including EHEC O157:H7 and O145:H28 strains, *Pseudomonas aeruginosa, Salmonella paratyphi*, *Vibrio* sp., *Klebsiella pneumoniae*, and *Acinetobacter baumannii* (22, 24). Using efficiency of plating (EOP) assays, Druantia III was shown to provide a broad range of defense against >70 phages (24).

Zorya defense systems are classified into Zorya types I, II, and III, which include a conserved ion-driven ZorA_5_B_2_ core complex containing the peptidoglycan-binding ZorB subunits and an elongated cytoplasmic tail assembled by the five ZorA subunits. The complex rotates and recruits different effector components for phage DNA degradation (20, 22, 25, 26). In Zorya II, the N-terminal helix of ZorB and the pentameric ZorA tail are shorter compared to Zorya I (26). The three Zorya subtypes also differ by their effector proteins. The effector of the Zorya II system was identified as the HNH endonuclease ZorE, which functions as a nickase, targeting phage and chromosomal DNA (26). Zorya II is distributed in *E. coli*, including EHEC O157:H7 and O145:H28 strains, as well as in other pathogenic proteobacteria, such as *Burkholderia* sp., *Vibrio* sp., *Serratia marcescens*, *Moraxella*, *Campylobacter*, *Legionella*, and *Pseudoalteromonas* sp. (22). Zorya II has been shown to protect against different phages, but the phage target spectra varied depending on the host bacteria (25, 26).

Moreover, prokaryotic immune systems were shown to often co-localize in so-called defense islands due to the integration of mobile genetic elements in distinct genomic hotspots (16, 17, 19, 22, 27, 28). The co-occurrence of phage defense systems in *E. coli* has been associated with their synergistic anti-phage activities, as demonstrated for the Zorya II and Druantia III defense systems (29). Synergistic immunity has been shown to provide an evolutionary advantage against host-specific phages at the population level (29). Recent studies in pathogenic antibiotic-resistant *P. aeruginosa* isolates revealed a correlation between the number of phage defense systems and their levels of phage resistance, indicating additive and synergistic interactions in the defense against the targeted phage spectra (30, 31).

In this work, we found that the EHEC strain EDL933 Δs*tx1/2* exhibits strong resistance toward many phages of the BASEL phage collection (32). DefenseFinder predicted three phage defense systems, which are encoded on O-island 172 (OI-172), including Zorya II, Druantia III, and an uncharacterized system, termed DISARM-related Antiviral Defense Array (ARMADA II), in work conducted in parallel to ours (33). This OI-172-associated phage defense island is distributed in O157:H7 strains and several non-pathogenic *E. coli* of the NCBI RefSeq collection. Using EOP and growth assays of single and combined defense system mutants, we analyzed the phage spectra and synergy of the three defense systems, revealing additive and synergistic interactions of Druantia III and ARMADA II compared to each system alone. Overall, our results validated a hitherto uncharacterized defense system in EHEC, ARMADA II, and demonstrated that the phage defense island on OI-172 is responsible for the strong phage resistance of EDL933 Δs*tx1/2*.

## RESULTS

### Identification of a phage defense island on OI-172 in EHEC O157:H7 strain EDL933

**Fig 1:**
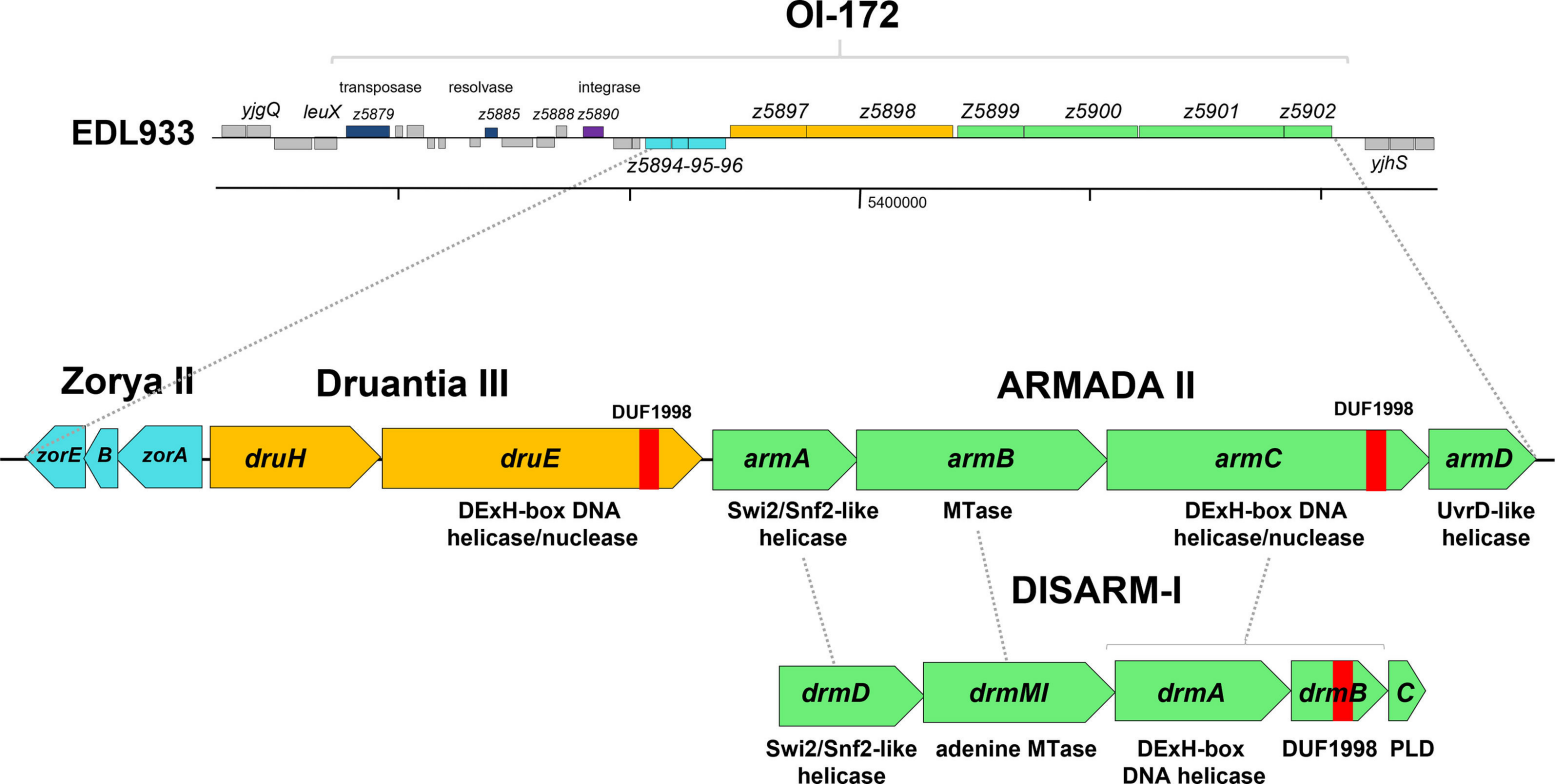
Gene organization of the Zorya II, Druantia III, and ARMADA II defense systems on OI-172 of EHEC O157:H7 strain EDL933 and comparison of the domain architectures to the DISARM I system. EDL933 encodes three phage defense systems on OI-172, i.e., Zorya II (*zorABE*), Druantia III (*druHE*), and ARMADA II (*armABCD*). The domain architectures of ARMADA II components, ArmA, ArmB, and ArmC, are related to those of DrmD, DrmMI, DrmA, and DrmB, respectively, of the DISARM I system, indicated by dashed lines. The two YprA-family DExH-box helicases/nucleases DruE and ArmC contain a DUF1998 and resemble a DrmAB fusion with an additional DrmC-homologous PLD domain (DruE) or two vsr-type and one EEP domain (ArmC). ARMADA II additionally encompasses a UvrD-like NTPase/helicase (ArmD) that resembles the GajB subunit of the Gabija system (34).

Using DefenseFinder (19), three phage defense systems were predicted to be encoded at the nine-gene locus *z5894*–*z5902* on OI-172 of *E. coli* O157:H7 strain EDL933, including Zorya II (ZorA, ZorB, and ZorE), Druantia III (DruH, DruE), and ARMADA II (ArmA, ArmB, ArmC, and ArmD) (33) (Fig. 1; Table S1). The Zorya II-encoding *zorABE* operon (*z5894*–*z5896*) is divergently transcribed from the Druantia III-encoding *druHE* operon (*z5897*–*z5898*) and the ARMADA II-encoding *armABCD* operon (*z5899*–*z5902*) (Fig. 1; Table S1). Based on their domain architectures, ArmC of ARMADA II and DruE of Druantia III both contain a DExH-box helicase of the YprA family (33), characterized by the presence of DUF1998. In the DISARM I system, the YprA-like helicase core and the DUF1998 domain are separated into two components, DrmA and DrmB, respectively, which form a stable complex (21). However, unlike DrmAB and DruE, ArmC carries two C-terminal vsr-type nuclease domains and a putative inactive endonuclease/exonuclease/phosphatase (EEP) domain (33).

Additionally, the predicted domain architecture of the ARMADA II components indicates further parallels to DISARM I. Akin to the DrmD and DrmMI components, ARMADA II also contains a RapA-like Swi2/Snf2-type NTPase/helicase (ArmA) and a putative adenine MTase (ArmB). While DrmMII and DrmC-like components are absent in ARMADA II, the system additionally includes a UvrD-like NTPase/helicase (ArmD), resembling the GajB subunit of the Gabija defense system (34) (Fig. 1; Table S1).

### Distribution of the OI-172 phage defense island across *E. coli* serotypes and pathotypes

KEGG homology analyses were used to investigate the distribution of the OI-172 phage defense island of *E. coli* EDL933 in bacteria. The genes encoding the defense systems Zorya II, Druantia III, and ARMADA II were found to co-localize mainly in atypical enteropathogenic *E. coli* (aEPEC) and STEC-aEPEC of serotypes O157:H7, O145:H28, and O55:H7, but also in the non-pathogenic antibiotic-resistant *E. coli* strains SMS-3-5 (35) and ATCC 8739 (Table S1).

**Fig 2:**
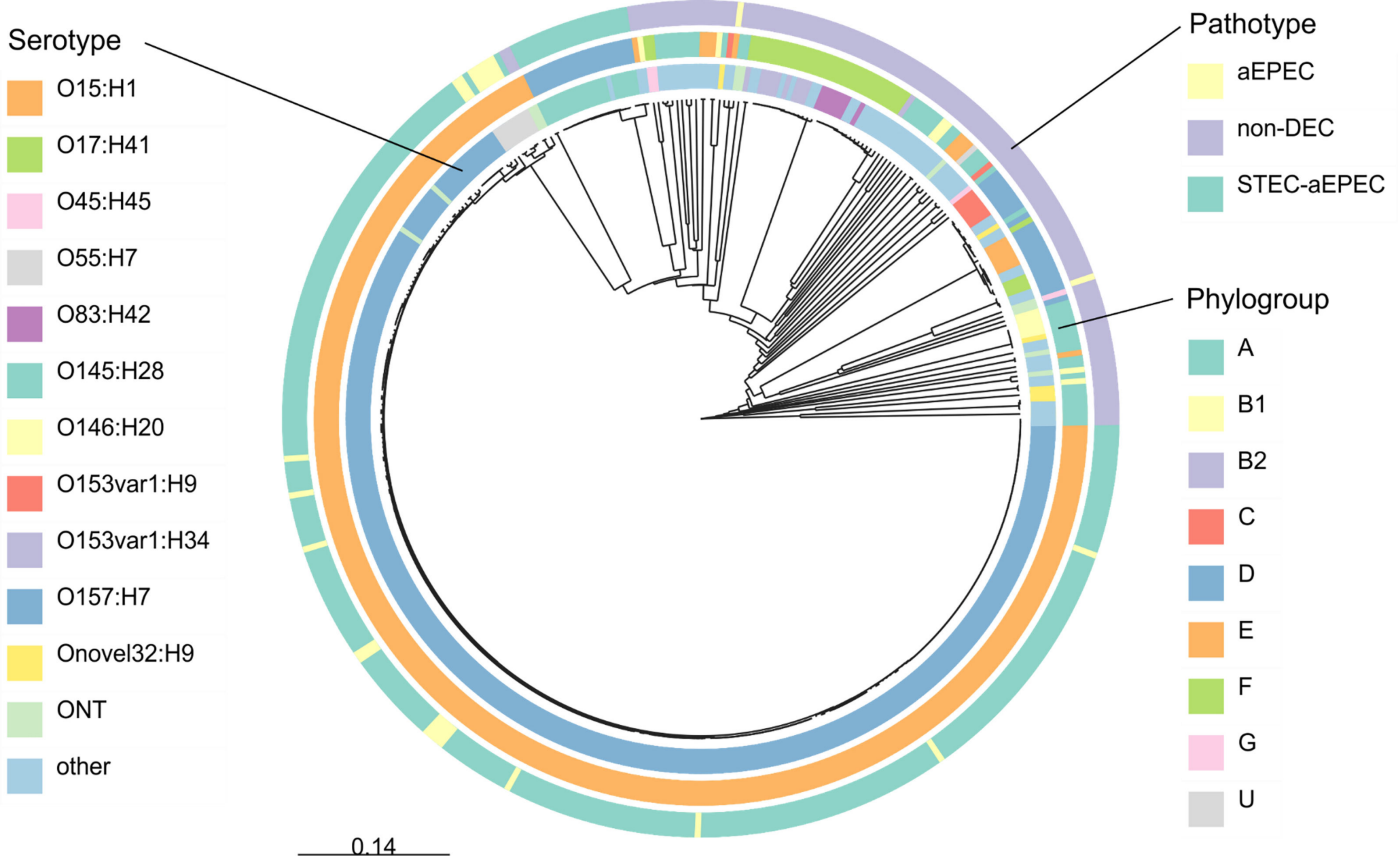
UPGMA tree of the co-localizing phage defense systems Zorya II, Druantia III, and ARMADA II across 424 *E. coli* genomes, including the STEC-aEPEC serotypes O157:H7, O55:H7, and O145:H28 and diverse non-pathogenic strains. The tree was calculated based on the distance matrices of extracted genome regions of the co-localizing Zorya II, Druantia III, and ARMADA II defense system-encoding genes as retrieved from *E. coli* genomes of the NCBI RefSeq database (Table S2) using mafft. Colors represent the strains’ characteristics, including serotypes (inner circle; only serotypes with at least three strains were represented; ONT, not typeable), phylogroups (middle circle), and pathotypes (outer circle; aEPEC, atypical enteropathogenic *E. coli*; STEC, Shiga toxin-producing *E. coli*; non-DEC, no diarrheagenic *E. coli*).

Next, we compared the genome sequence encoding the three defense systems of strain EDL933 against the NCBI RefSeq database, covering 3,792 complete *E. coli* genome sequences (36). The results revealed the occurrence of the three co-localizing defense systems Zorya II, Druantia III, and ARMADA II in 426 *E. coli* strains, including non-pathogenic commensal *E. coli* and pathogenic aEPEC and STEC-aEPEC strains of various Clermont phylogroups comprising 66 distinct serotypes (Table S2). The diversity of the complete defense island across the 426 *E. coli* genomes is mostly based on variations of the *armB* gene, whereas other components of the three defense systems are highly conserved (up to 100% identity) as displayed in the phylogenetic tree (Table S2; Fig. 2).

The three defense systems were most prevalent in 277 STEC-aEPEC O157:H7 strains of phylogroup E, and also detected in STEC-aEPEC/aEPEC O55:H7 (*n* = 8) and O145:H28 (*n* = 19) strains (Table S2; Fig. 2). The genes for the three defense systems also co-localize in the genomes of 117 non-pathogenic *E. coli* of highly diverse phylogroups and serotypes, such as O15:H1, O17:H41, O45:H45, O83:H42, O146:H20, O153var1:H9/H34, and Onovel32:H9 as the most prevalent ones (Table S2; Fig. 2). The bioinformatics analyses revealed that the defense islands show a lower degree of sequence conservation for the *armB* gene in non-pathogenic strains, forming diverse phylogenetic clusters distant from the O157:H7/O55:H7 and O145:H28 lineages (EHEC1 clonal complex) (37) (Table S2; Fig. 2). Overall, our data indicate that the defense island is distributed across many *E. coli* serotypes, phylogroups, and lineages, but occurs most frequently and conserved in STEC-aEPEC of the EHEC1 clonal complex. Interestingly, other important human-pathogenic STEC/EHEC serotypes, such as O26:H11, O103:H2, O111:H8/H11, or O118:H16 (EHEC2 clonal complex) (37), completely lack the OI-172-associated defense island.

The data suggest that the defense island has possibly co-evolved with specific *E. coli* serotypes, such as the O157/O55/O145 lineages. The ABRicate sequence comparisons of the single genes of the three defense systems and the fastANI analyses of the complete defense locus (*zorE* to *armD*) showed high similarities compared to the reference sequence of strain EDL933 (Table S2), supporting this hypothesis. However, further ABRicate analyses of the defense locus (*zorE* to *armD*) and the whole OI-172 (*z5878* to *z5904*) showed higher variability of the whole island compared to the individual defense genes of the three systems, especially across non-pathogenic *E. coli* strains, but even within the O157:H7 and O55:H7 strains (Table S2). This variability of the OI-172 across *E. coli* and *Salmonella* strains was already described previously (38).

### The EHEC strain EDL933 Δ*stx1/2* is resistant to many phages of the BASEL phage collection

**Fig 3:**
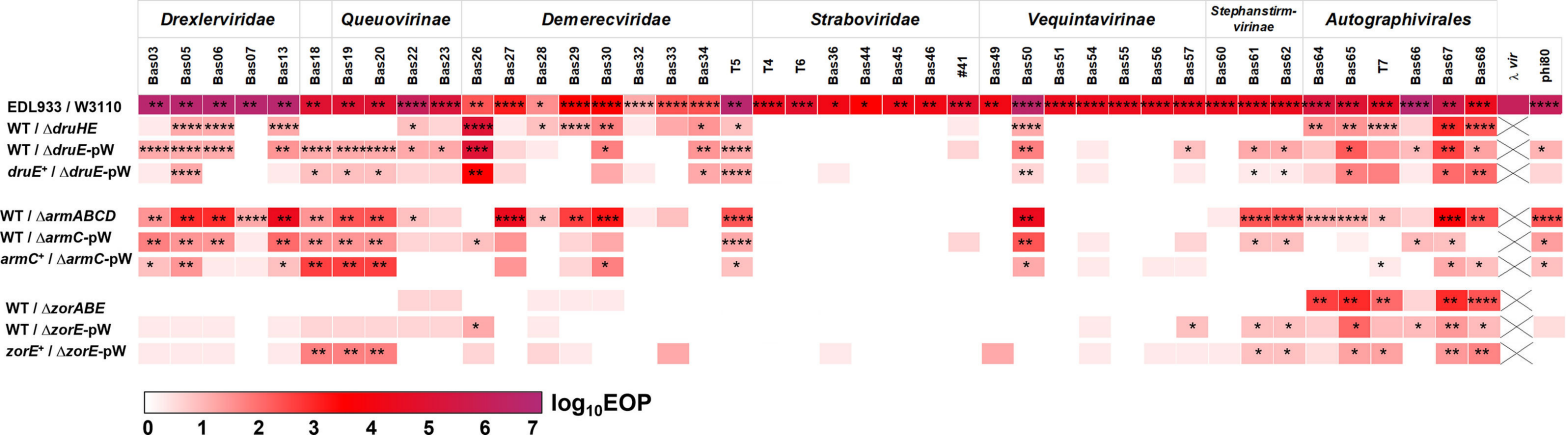
The OI-172-encoded Zorya II, Druantia III, and ARMADA II defense systems confer resistance against a broad phage spectrum in strain EDL933 Δ*stx1/2*. Heatmap showing protection by the defense systems against a spectrum of 44 phages. First, the phage resistance of EHEC versus the sensitive *E. coli* W3110 was determined in PFU/EOP assays. Second, the log_10_ fold-changes in phage defense were determined using PFU/EOP assays of the 44 phages in the EDL933 Δ*stx1/2* (WT) strain versus the Δ*zorABE*, Δ*druHE*, or Δ*armABCD* deletion mutants. Third, PFU/EOP assays were used to validate the complementation of the Δ*zorE*, Δ*druE*, and Δ*armC* effector mutants with pWKS30-encoded z*orE*, *druE*, *and armC*, respectively (z*orE^+^*, *druE^+^*, and *armC^+^*), versus the mutants with empty pWKS30 plasmids (Δ*zorE-*pW, Δ*druE*-pW*,* Δ*armC*-pW), respectively. Log_10_ EOP values are averages from 3 to 4 biological replicate experiments, which are presented in Tables S3 to S5. The red-magenta color code indicates the scale of log_10_ EOP values. The statistics for the log_10_ EOP values were calculated for log_10_ PFU WT/defense mutants or log_10_ PFU complemented strains/defense mutants for each phage using Student’s unpaired one-tailed *t*-test of two samples of unequal variance. *P* values: *, *P* < 0.05; **, *P* < 0.01; ***, *P* < 0.001; ****, *P* < 0.0001. No plaques were detected for λ*vir*.

We hypothesized that the three OI-172-encoded phage defense systems confer a broad range of phage resistance in the EHEC strain EDL933. Plaque-forming unit (PFU) and EOP assays were used to determine the phage susceptibility of the EHEC strain EDL933 Δ*stx1/2* PFU versus the *E. coli* K-12 strain W3110 against 44 *E. coli* phages of the BASEL phage collection (32), belonging to the *Drexlerviridae*, *Queuovirinae*, *Demerecviridae*, *Straboviridae*, *Vequintavirinae*, *Stephanstirmvirinae*, and *Autographivirales* (Fig. 3; Table S3). The results revealed significant EOP differences of up to 10^7^-fold for the EHEC strain versus the W3110 strain for the 44 phages, indicating that the EHEC strain is highly resistant to many phages (Fig. 3; Table S3). The EHEC strain EDL933 Δ*stx1/2* exhibited complete resistance against *Drexlerviridae*, *Straboviridae*, *Vequintavirinae*, *Stephanstirmvirinae*, and *Autographivirales*. However, the EHEC strain was more sensitive toward phages of the *Demerecviridae* (Bas26, Bas27, Bas28, Bas29, Bas30, Bas32, Bas33, and Bas34), with significant EOP differences of 10^2^- to 10^3^-fold compared to *E. coli* W3110, indicating that *Demerecviridae* are successful anti-EHEC phages. Altogether, our results clearly point to strong resistance of the EHEC strain EDL933 Δ*stx1/2* toward a broad range of different phage families.

### The Zorya II, Druantia III, and ARMADA II systems on OI-172 confer protection against a broad range of phages

Next, we constructed Δ*zorABE*, Δ*druHE*, or Δ*armABCD* system deletion mutants, the Δ*zorE*, Δ*druE*, and Δ*armC* helicase/nuclease effector mutants, as well as the *zorE*, *druE*, and *armC* complemented strains in the background of *E. coli* EDL933 Δ*stx1/2*. EOP assays were used to analyze the phage susceptibility of the defense mutants against the panel of 44 BASEL phages (32) in comparison to *E. coli* EDL933 Δ*stx1/2*, which is referred to as wild type (WT) (Fig. 3; Table S4).

The EOP assays of the Δ*zorABE*, Δ*druHE*, and Δ*armABCD* mutants revealed that the three defense systems contribute to different extents to the defense against phages. Specifically, the Δ*zorABE* mutant exhibited significantly (10^2^- to 10^3^-fold) enhanced sensitivity toward the *Autographivirales*, but no significantly augmented sensitivity against other phages (Fig. 3; Table S4). The EOP assays of the Δ*zorE* mutant and the pWKS30-*zorE* complemented strain confirmed the significant protection of Zorya II against *Autographivirales*, but showed also significant defense against *Dhillonvirus* (Bas18), *Queuovirinae* (Bas19, Bas20), and *Stephanstirmvirinae* (Bas61, Bas62) upon plasmid-based production (Fig. 3; Table S5). These results indicate that Zorya II protects significantly against the *Autographivirales* in the native host strain, but can provide additional immunity against other phages upon overexpression.

Deletion of Druantia III resulted in significantly (10- to 10^2^-fold) enhanced susceptibility toward phages of the *Drexlerviridae* (Bas5, Bas6, Bas13), *Queuovirinae* (Bas22), *Demerecviridae* (Bas28, Bas29, Bas30, Bas34, T5), and *Vequintavirinae* (Bas50) (Fig. 3; Table S4). Additionally, the Δ*druHE* mutant exhibited the strongest (10^2^- to 10^4^-fold) sensitivity against the *Autographivirales* and Bas26. The EOP assays of the Δ*druE* mutant and the pWKS30-*druE* complemented strains confirmed the pattern of phage protection as observed for the Δ*druHE* mutant (Fig. 3; Table S5). These results indicate that Druantia III confers significant resistance against a broad spectrum of phages of various phage families, with the strongest protection against Bas26 and *Autographivirales*.

Interestingly, the ARMADA II-deficient Δ*armABCD* mutant showed significant sensitivity against a similar spectrum of phages of the *Drexlerviridae*, *Queuovirinae*, *Demerecviridae*, *Vequintavirinae*, *Stephanstirmvirinae*, and *Autographivirales*, as the Δ*druHE* mutant (Fig. 3; Table S4). Moreover, the Δ*armABCD* mutant exhibited significantly (10^2^- to 10^5^-fold) higher phage sensitivity against the *Drexlerviridae*, *Dhillonvirus* (Bas18), *Queuovirinae* (Bas19, Bas20), *Demerecviridae* (Bas27, Bas29, Bas30, T5), *Vequintavirinae* (Bas50), and *Stephanstirmvirinae* (Bas61, Bas62), whereas Druantia III conferred only weak protection against these phages (Fig. 3; Table S4). Furthermore, the EOP assays revealed a similar (10^2^- to 10^4^-fold) higher phage sensitivity of both Δ*armABCD* and Δ*druHE* mutants against the *Autographivirales* (Fig. 3; Table S4). EOP assays with the Δ*armC* mutant confirmed the spectrum of sensitive phages as observed in the Δ*armABCD* mutant (Fig. 3; Table S5). Complementation of the Δ*armC* mutant with pWKS30-*armC* restored phage resistance, albeit at a lower level than that observed for the WT, indicating incomplete complementation with ArmC produced ectopically (Fig. 3; Table S5). Overall, our results indicate that ARMADA II provides the strongest level of immunity against a broad spectrum of phages, as compared to Zorya II and Druantia III, while each of these defense systems similarly contributes to resistance toward *Autographivirales*. However, none of these nuclease-based defense systems confers protection against the *Straboviridae* (Fig. 3; Table S4), which are known to have hypermodified phage genomes (39, 40). Thus, consistent with their resistance against DNA-targeting RM systems (32), *Straboviridae* also showed complete resistance against Zorya II, Druantia III, and ARMADA II of this EHEC-associated phage defense island.

### The individual components of the Zorya II, Druantia III, and ARMADA II systems are essential for the full anti-phage defense

**Fig 4:**
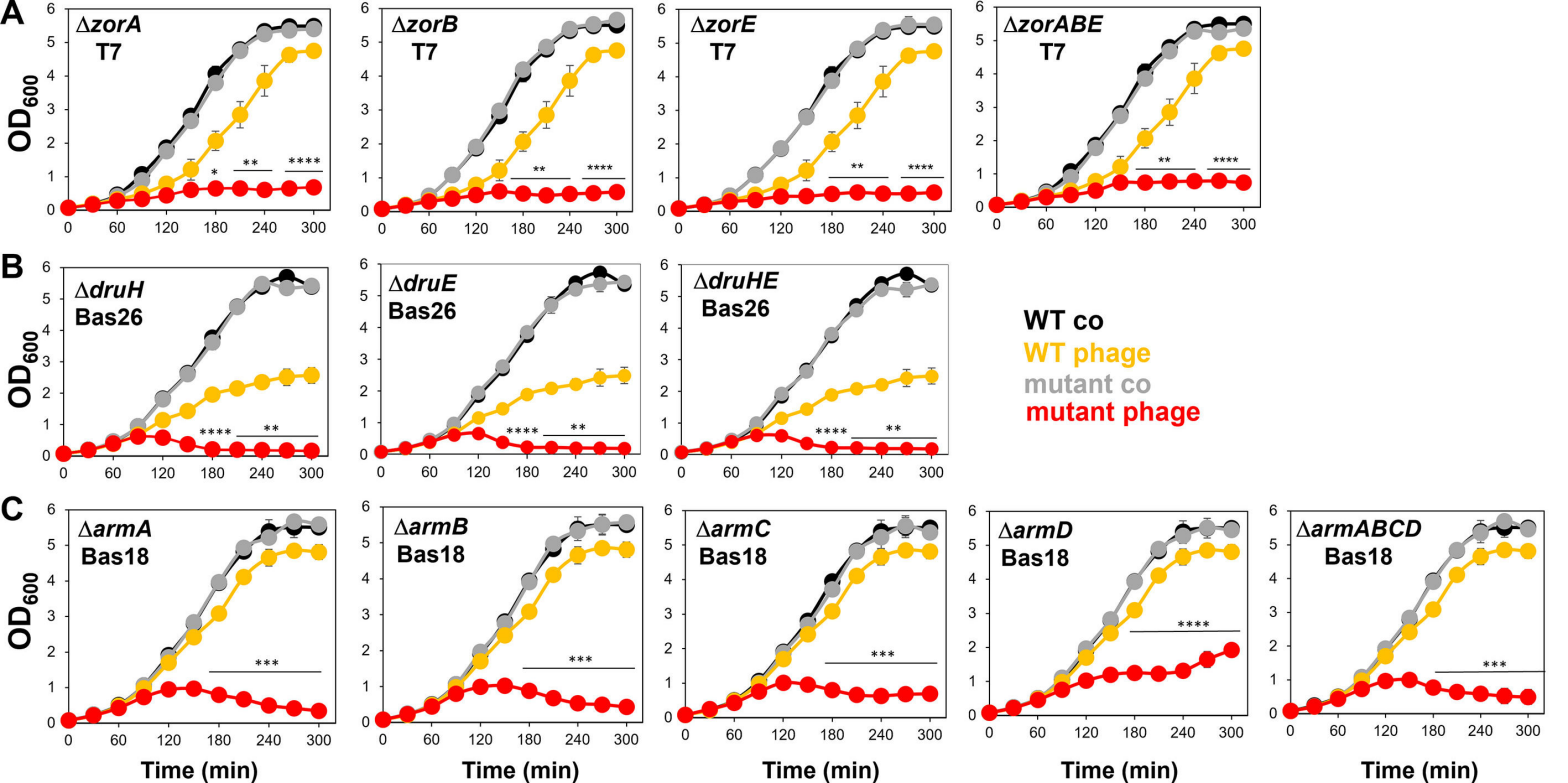
The individual components of the Zorya II, Druantia III, and ARMADA II systems are essential for the defense against sensitive phages (T7, Bas26, Bas18, respectively). Growth curves of the strain EDL933 Δ*stx1/2* (WT) versus (**A**) the Δ*zorA*, Δ*zorB*, Δ*zorE*, and Δ*zorABE* mutants after infection with phage T7 (MOI 7), (**B**) the Δ*druH*, Δ*druE*, and Δ*druHE* mutants after Bas26 infection (MOI 3), and (**C**) the Δ*armA*, Δ*armB*, Δ*armC*, Δ*armD*, and Δ*armABCD* mutants, infected with Bas18 (MOI 7). The EHEC strains were grown in LB until an OD_600_ of 0.08, left untreated as a control (black, WT; gray, defense mutant), or subjected to phage infection (yellow, WT; red, defense mutant) to monitor the growth curves of the bacterial cultures. The OD_600_ values are average values from three biological replicates, and error bars represent the standard deviations (SD). The statistics for the OD_600_ values at different time points of the growth curves of the phage-infected WT versus the mutants were calculated using Student’s unpaired two-tailed *t*-test of two samples of unequal variance. *P* values of infected WT versus mutants indicate: *, *P* < 0.05; **, *P* < 0.01; ***, *P* < 0.001; ****, *P* < 0.0001.

Next, we were interested in whether the individual components of the Zorya II, Druantia III, and ARMADA II systems are required for the specific anti-phage defense. Thus, we monitored the growth of the individual defense mutants in liquid cultures after infection with susceptible phages for each defense system (T7, Bas26, or Bas18) at specific multiplicities of infection (MOI) (Fig. 4A, B, and C).

In growth assays, the Δ*zorA*, Δ*zorB*, Δ*zorE*, and Δ*zorABE* mutants were all sensitive toward infection with phage T7 (MOI 7), while the WT was resistant (Fig. 4A). These data confirmed previous findings that ZorA, ZorB, and ZorE are essential for the Zorya II-mediated anti-phage defense (22).

Similarly, the cultures of the Δ*druH*, Δ*druE*, and Δ*druHE* mutants collapsed after infection with Bas26 (MOI 3), whereas the infected WT was able to grow until an OD_600_ of 2.5 (Fig. 4B). These results indicate that DruH and DruE are essential for Druantia III anti-phage defense.

After infection of the Δ*armA*, Δ*armB*, Δ*armC*, Δ*armD*, and Δ*armABCD* mutants with Bas18 (MOI 7), the strains were unable to grow and lysed, but the growth of the WT was unaffected (Fig. 4C). Of note, the growth of the Δ*armD* mutant was slightly increased at later time points after Bas18 infection, but was still significantly different from the WT. These results support that ArmA, ArmB, ArmC, and ArmD are all essential for the ARMADA II anti-phage defense.

### Additive and synergistic interactions of Zorya II, Druantia III, and ARMADA II in the anti-phage defense of the EHEC strain EDL933 Δ*stx1/2*

**Fig 5:**
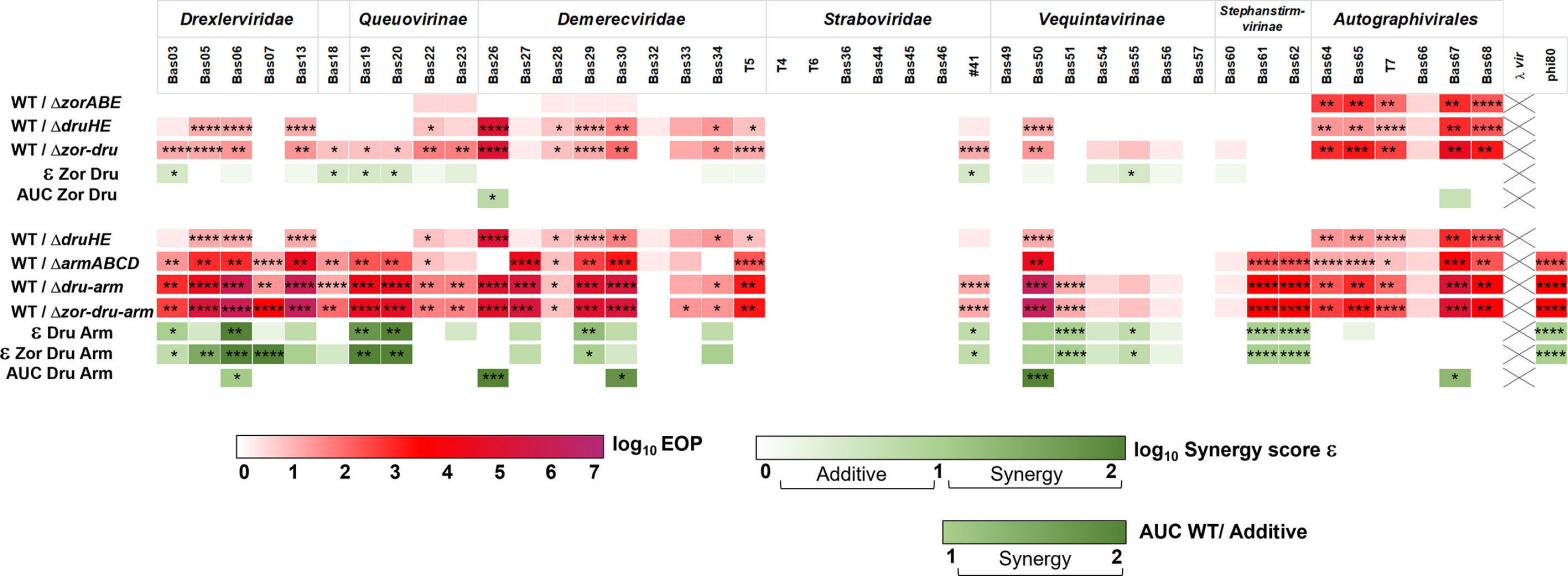
Druantia III and ARMADA II provide additive and synergistic protection against a broad phage spectrum. The heatmap shows the log_10_ EOP values of 44 phages in the Δ*zorABE*, Δ*druHE*, and Δ*armABCD* single and Δ*zor-dru*, Δ*dru-arm*, and Δ*zor-dru-arm* combined defense system mutants quantified versus the *E. coli* EDL933 Δ*stx1/2* (WT). Log_10_ EOP values are averages from four biological replicate experiments, which are presented in Table S4. The synergy score was calculated as described in the Materials and Methods, indicating the epistatic coefficient ε as the log_10_ higher EOP values of the combined Δ*zor-dru*, Δ*dru-arm*, or Δ*zor-dru-arm* mutants above the calculated additive protection by the single system mutants. Synergy was defined when log10 EOP values of the combined mutants were above the additive log10 EOP values (>additive +1) of the individual defense mutants. The statistics for significant synergistic or additive interactions were calculated using Student’s unpaired one-tailed *t*-test of two samples of unequal variance (Welch’s *t*-test) for the log10 EOP of combined defense mutants (Δ*zor-dru*, Δ*dru-arm*, or Δ*zor-dru-arm*) versus the additive log10 EOP values of the single system mutants (Δ*zorABE*, Δ*druHE*, Δarm*ABCD*) and are presented in Table S4. *P* values: *, *P* < 0.05; **, *P* < 0.01; ***, *P* < 0.001; ****, *P* < 0.0001. Additionally, we included the summary from the AUC calculation (Fig. S1 and S2) to visualize synergistic and additive interactions of Zorya II/Druantia III and Druantia III/ARMADA II against Bas6, Bas26, Bas30, Bas50, and Bas67 using the ratio of WT AUC versus additive AUC of single system mutants. Statistics of synergistic interactions are calculated as described in Fig. S2.

The co-occurrence of the Zorya II, Druantia III, and ARMADA II defense systems suggests their synergistic interactions and complementary activities in the anti-phage defense in the EHEC strain EDL933 Δ*stx1/2*. To test this hypothesis, we deleted the co-localizing defense system pairs Zorya II/Druantia III and Druantia III/ARMADA II, as well as the three systems Zorya II/Druantia III/ARMADA II in strain EDL933 Δ*stx1/2*. Using EOP assays, the phage sensitivities of the Δ*zor-dru*, Δ*dru-arm*, and Δ*zor-dru-arm* combined system mutants were analyzed against the panel of 44 phages (Fig. 5; Table S4). To assess synergistic interactions, the epistatic coefficient ε was calculated as a synergy score as in previous studies (29). Systems were considered to act synergistically when the EOP values of the combined system mutant (e.g., Δ*zor-dru*, Δ*dru-arm*, and Δ*zor-dru-arm*) significantly exceeded the sum of the anti-phage effects of the single defense mutants by a factor of log_10_ >1, which is displayed as a green color code in the heatmap (Fig. 5; Table S4).

The EOP assays showed that Zorya II and Druantia III act significantly additively against six phages (Bas03, Bas06, Bas18, Bas19, Bas20, #41, and Bas55), since the epistatic coefficient of the Δ*zor-dru* mutant was below log_10_ of 1 compared to the sum of the EOP values of the Δ*zorABE* and Δ*druHE* mutants (Fig. 5; Table S4). While the Δ*zorABE* and Δ*druHE* mutants showed significantly enhanced sensitivity toward *Autographivirales*, the sensitivity of the Δ*zor-dru* mutant was not significantly higher compared to each single system mutant (Fig. 5; Table S4). These results indicate that Zorya II and Druantia III showed only additive protection against a few phages, but not against *Autographivirales*, which are efficiently targeted by both individual systems.

The deletion of Druantia III and ARMADA II resulted in significant hypersensitivity toward 11 phages, including *Drexlerviridae*, *Dhillonvirus*, *Queuovirinae*, *Demerecviridae*, *Vequintavirinae*, and *Stephanstirmvirinae* (Fig. 5; Table S4). The epistatic coefficient of the Δ*dru-arm* mutant exceeded values of log_10_ >1 over the additive EOP of the Δ*druHE* and Δ*armABCD* mutants for four phages (Bas6, Bas19, Bas20, and Bas29), supporting significant synergistic interactions of Druantia III and ARMADA II. Furthermore, Druantia III and ARMADA II showed significant additive protection against seven phages (Bas3, Bas41, Bas51, Bas55, Bas61, Bas62, and Phi80), since the epistatic coefficient of the Δ*dru-arm* mutant was lower than log_10_ of 1 compared to the additive EOP values of the Δ*druHE* and Δ*armABCD* mutants. In the Δ*zor-dru-arm* full defense mutant, we observed significantly synergistic defenses against five phages (Bas5, Bas6, Bas7, Bas19, and Bas20), as well as additive interactions against eight phages (Fig. 5; Table S4). Taken together, the EOP assays indicate that Druantia III and ARMADA II act additively and synergistically in the defense against a broad spectrum of phages, while Zorya II and Druantia III interact additively in the defense against a few phages.

To validate the EOP results, we performed liquid growth experiments of the WT and defense mutants, which were infected with Bas6, Bas20, Bas26, Bas27, Bas30, Bas50, and Bas67 as the most susceptible phages for the three defense systems (Fig. 5; Fig. S1 and S2). Synergistic effects of the defense pairs were considered when the calculated AUC from the growth curve of the WT with the three defense systems was significantly higher than the additive AUC of the single defense system mutants.

The growth experiments showed that the AUC values of the WT were lower than the additive AUC values of the Δ*zorABE* and Δ*druHE* mutants after infection by Bas6, Bas20, Bas27, Bas30, and Bas50, indicating no additive or synergistic interactions of Zorya II and Druantia III (Fig. S1 and S2). However, Zorya II and Druantia III showed weak synergistic and additive interactions against Bas26 (MOI 0.05) and Bas67 (MOI 7), respectively, since the AUC values of the WT curves were higher for Bas26 and in a similar range for Bas67 compared to the additive AUC of the Δ*druHE* and Δ*zorABE* mutants. Thus, the growth experiments identified additional interactions for Zorya II and Druantia III, not observed in the EOP assays (Fig. 5; Fig. S1 and S2).

The growth experiments further revealed significant synergistic interactions of Druantia III and ARMADA II against Bas6, Bas26, Bas30, Bas50, and Bas67, since the AUC values of the WT curves were significantly higher than the additive AUC of the Δ*druHE* and Δ*armABCD* mutants (Fig. 5; Fig. S1 and S2). Thus, the EOP and growth experiments are complementary and identified synergistic interactions of the Druantia III and ARMADA II against eight phages, including four phages only found using the more sensitive growth assays (Fig. 5; Fig. S1 and S2).

However, the growth experiments did not allow evaluation of the synergy of the three defense systems, Zorya II, Druantia III, and ARMADA II, since the additive AUC of the three single defense mutants exceeded the AUC value of the WT, due to the high phage resistance of the Δ*druHE* and Δ*zorABE* mutants (Fig. S1 and S2). Thus, we compared the AUC values of the Δ*zor-dru-arm* and Δ*dru-arm* mutants and found that the absence of all three defense systems leads to significantly enhanced sensitivity against seven phages (Bas6, Bas20, Bas26, Bas27, Bas30, Bas50, and Bas67), compared to the combined loss of Druantia III and ARMADA II (Fig. S2). These data support the significant contribution of Zorya II to the defense against a wider spectrum of phages in the EHEC strain, which was not observed in the EOP assays (Fig. 5; Fig. S1 and S2). Overall, our EOP and growth results revealed significant additive and synergistic interactions of Druantia III and ARMADA II against a broad spectrum of phages, whereas Zorya II and Druantia III act additively against a few phages in the EHEC strain EDL933 Δ*stx1/2*.

## DISCUSSION

In this manuscript, we discovered an OI-172-associated phage defense island of EHEC strain EDL933 Δ*stx1/2*, which encodes the nuclease-based defense systems, Zorya II, Druantia III, and ARMADA II. This phage defense island is most prevalent in the STEC-aEPEC serotypes O157:H7, O145:H28, and O55:H7 (EHEC1 clonal complex), which are evolutionarily related (41–44). The ABRicate analyses of individual defense genes and the fastANI analysis of the complete defense locus (*zorE* to *armD*) revealed a high conservation of the three phage defense systems across the O55:H7/O157:H7/O145:H28 lineage, suggesting that the evolution of the phage defense island is most likely related to this lineage (Table S2). However, sequence comparison of the whole OI-172 revealed a high variability of this island across non-pathogenic *E. coli* and the O55:H7/O157:H7/O145:H28 lineage (38), indicating that the acquisition of the three phage defense systems *via* horizontal gene transfer of mobile genetic elements, e.g., via phages, cannot be excluded. This is further supported by the presence of putative integrase, transposase, and resolvase genes in the OI-172 of various O157:H7 strains as noted previously (38). Since the STEC-aEPEC O157/O55/O145 serotypes carry up to 18 prophages and prophage-like elements (41–44), this phage defense island might have evolved together with their encountered phage foes, which were integrated into the genomes as lambdoid prophages. However, other more prevalent STEC EHEC serogroups, such as O26, O103, O111, or O118 of the EHEC2 clonal complex, do not harbor the phage defense island and might possibly encode alternative phage defense systems (44) (Table S2).

Previous studies revealed a correlation of the number of defense systems with phage resistance phenotypes in clinical and antibiotic-resistant *P. aeruginosa* isolates (30, 31). Here, we report that the EHEC outbreak strain EDL933 Δ*stx1/2* is highly resistant toward many phage families, including *Drexlerviridae*, *Queuovirinae*, *Demerecviridae*, *Straboviridae*, *Vequintavirinae*, *Stephanstirmvirinae*, and *Autographivirales*. However, the EHEC strain was less resistant toward some *Demerecviridae*, supporting previous findings that O157:H7 strains were efficiently lysed by *Epseptimaviruses*, which bind to the BtuB outer membrane protein as a secondary host cell receptor (15, 32, 45, 46). It was demonstrated that the *Demerecviridae* are resistant to most bacterial nuclease-based types I/II/III restriction-modification systems, and inhibit host-mediated methylation, possibly explaining why *Demerecviridae* are more efficient anti-EHEC phages (47).

In this study, we found that the three co-localizing Zorya II, Druantia III, and ARMADA II defense systems on OI-172 are responsible for mediating the strong phage resistance of EHEC strain EDL933 Δ*stx1/2*, since deletion of the three systems sensitizes the EHEC strain EDL933 Δ*stx1/2* to the majority of tested phages. Thus, the OI-172 phage defense island might be crucial for the survival of EHEC upon interaction with phages in the ecological context.

We also investigated the phage spectra and the synergy of the three defense systems in the native host strain EHEC EDL933 Δ*stx1/2*. The EOP results of the Δ*zorABE* mutant showed that Zorya II confers significant protection against *Autographivirales*, but not against other phage families. These results confirm previous findings of plasmid-expressed Zorya II in *E. coli* strains MG1655 and MT56, providing strong immunity against phage T7 (22, 25, 26). However, Zorya II systems from different bacteria varied strongly in their phage target spectra when expressed in *E. coli* MT56, indicating that the Zorya II target phages also depend on the bacterial host (22, 25, 26). Thus, the limited phage spectrum of Zorya II in the EHEC strain EDL933 might be related to the host background and its observed phage resistance.

Moreover, T7 phages and Zorya II rely on an intact PMF for phage DNA translocation and the corresponding phage defense by rotation of the pentameric ZorA tail for ZorE effector recruitment (48, 49), possibly explaining why *Autographivirales* are strong targets for Zorya II in *E. coli* strains EDL933, ATCC 8739, and KTE66 (22, 25, 26).

Furthermore, Druantia III was shown to protect weakly but significantly against 15 phages of the *Drexlerviridae*, *Queuovirinae*, *Demerecviridae*, *Vequintavirinae*, and *Autographivirales* in the EHEC strain EDL933 Δ*stx1/2.* Previously, strong protection against >35 phages was found for heterologously expressed Druantia I and Druantia III in *E. coli* MG1655 (22, 24). These data indicate that the heterologous expression of Druantia III leads to higher levels of phage defense compared to that observed in the native host strain.

We also explored the role of the ARMADA II system, which provided much stronger protection levels as compared to Druantia III against a similar phage spectrum, including *Drexlerviridae*, *Dhillonvirus*, *Queuovirinae*, *Demerecviridae*, *Vequintavirinae* (Bas50), *Stephanstirmvirinae*, and *Autographivirales*. Additionally, ARMADA II showed specific and strong defense against Bas27, whereas Bas26 was identified as a strong and specific target for Druantia III. Thus, beyond their similar phage family targets, both systems also act specifically against selected phages, indicating additive activities that result in a broader phage spectrum.

Moreover, the EOP and growth assays revealed significantly additive defenses of Zorya II and Druantia III against seven phages of the *Drexlerviridae*, *Dhillonvirus*, *Queuovirinae*, *Demerecviridae*, *Vequintavirinae*, and *Autographivirales*. Previously, Druantia III and Zorya II systems were shown to act synergistically against phages T1 and T3 when expressed in an *E. coli* K-12 laboratory strain (29). The weaker additive interactions in our work might be explained by the analysis of defense mutants in the EHEC strain, which is more resistant to many phages compared to *E. coli* K-12 strains (29).

Importantly, our EOP and growth experiments showed significantly synergistic and additive interactions of Druantia III and ARMADA II in the defense against a broad spectrum of phages of different families, together providing more robust immunity than the individual systems alone in the native host. This observation raises the question about the molecular mechanisms of the interaction of both systems in the phage defense. Previously, the molecular basis of the synergistic interactions between Gabija and tmn has been investigated, showing that tmn co-opted the ATPase domain of Gabija for an improved phage defense (29). Similarly, Druantia III and ARMADA II share effectors with common domains, such as the YprA-family DExH box NTPases/helicases (DruE and ArmC) with a DUF1998 domain and different C-terminal domains (PLD-like and a vsr-type nuclease, respectively), resembling fused DrmABC components of DISARM systems. Thus, DruE and ArmC could cooperate in the translocation or degradation of the phage DNA for improved phage defense. Alternatively, the putative helicase/nuclease activities provided by DruE and ArmC might also function in the context of the accessory factors of the respective other system.

Due to their related domain architectures, we hypothesize that DruE of Druantia III, ArmC of ARMADA II, and the combined DrmA, DrmB, and DrmC proteins of DISARM systems might function via a similar mode of action during the anti-phage defense. In agreement with the target phages of Druantia III and ARMADA II, the DISARM I and II systems also provide protection against a broad spectrum of phages (21, 50, 51). It was proposed that DISARM systems recognize single-stranded overhangs of phage DNA substrates but not sequence-specific motifs to circumvent immune evasion mechanisms of phages by escape mutations and to avoid autoimmunity (21). Based on their broad spectra of target phages, we anticipate that DruE and ArmC also recognize the structural context of the DNA substrate but do not degrade phage DNA in a sequence-specific manner.

The Zorya II, Druantia III, and ARMADA II systems did not provide immunity against *Straboviridae*, which are known for their hypermodified phage genomes, including 5-hydroxymethylcytosines (hmCs) and 5-hydroxymethylglucosylcytosines (ghmCs) for *Tequatrovirus* and 5-hydroxymethyl-arabinosylcytosines for *Mosigvirus* (39, 40). The roles of phage DNA modifications for several nuclease-based defense systems have been studied using phage T4 mutants deficient in hmC and ghmC modifications (24). PFU assays showed that the presence of phage T4 DNA modifications abolished the Druantia III-mediated defense *in vivo* (24). Thus, Druantia III and ARMADA II most likely recognize unmodified phage DNA. Our current studies are directed at elucidating the molecular mechanisms of the Druantia III and ARMADA II-based anti-phage defense.

## MATERIALS AND METHODS

### Bioinformatics analysis for the distribution of the OI-172 defense island in *E. coli*

Public data were investigated concerning the co-occurrence of the genes encoding the three phage defense systems Zorya II, Druantia III, and ARMADA II in pathogenic and non-pathogenic *E. coli* strains. Therefore, all available complete *E. coli* genome sequences (*n* = 3,792) from NCBI RefSeq (https://www.ncbi.nlm.nih.gov/refseq/, accessed on 6 March 2025) (36) were retrieved. The presence of single genes was determined using ABRicate (https://github.com/tseemann/abricate, with mincov = 50, minid = 15) against a custom database of single defense genes of the gene locus *z5894* to *z5902* from EHEC strain EDL933 (NCBI RefSeq assembly GCF_000006665.1).

Based on the results, only strains comprising at least one complete defense system of the OI-172 defense island (i.e., Zorya II, Druantia III, or ARMADA II) were included in further analyses (*n* = 467, Table S2). Strains were characterized using BakCharak (https://gitlab.com/bfr_bioinformatics/bakcharak), which implements the determination of the genoserotype based on the CGE SeroTypeFinder (52) and the EcOH database (53), as well as the MLST typing tool mlst (https://github.com/tseemann/mlst), the ClermontTyping for determination of the phylogroup (https://github.com/A-BN/ClermonTyping) (54), the virulence finder database (55), and AMRFinderPlus v4.0 (56) for determination of the *E. coli* pathotype. Sequence annotation was performed using bakta (57).

Sequence extraction was performed using the extract_genes_ABRicate script (https://github.com/boasvdp/extract_genes_ABRicate) based on the results of an ABRicate analysis comprising the region of the three defense systems (*z5894* to *z5902*) from the EHEC strain EDL933. A distance matrix and the respective UPGMA tree were calculated using mafft (options: --retree 0 –treeout, https://mafft.cbrc.jp/alignment/software/treeout.html). Tree visualization was done using https://microreact.org/. For sequence comparisons of the three defense systems, only 424 strains with all nine genes of the OI-172-associated defense island were used from the first ABRicate analysis. Two sequences were excluded due to an unusual length of the extracted sequence (GCF_017165395.1) and a duplication in the ARMADA II system (GCF_016942635.1). Besides the sequence comparison, an average nucleotide identity (ANI) analysis using fastANI (https://github.com/ParBLiSS/FastANI) was performed to provide information on the nucleotide identity of the genomic region ranging from *z5894* to *z5902*, encoding the three defense systems (Table S2). Furthermore, an additional ABRicate analysis was performed using the whole OI-172 defense island (gene locus *z5878* to *z5904* from EHEC strain EDL933 NCBI RefSeq assembly GCF_000006665.1, with mincov = 50, minid = 15) to show the higher variability of the whole OI-172 defense island compared to that of the cluster of the three defense systems (Table S2).

### Bacterial strains and cultivation

All bacterial strains, plasmids, phages, and primers used in this work are listed in Tables S6, S7, and S8. *E. coli* strain DH5α was used as a cloning host for plasmid constructions. Additionally, *E. coli* K-12 strains W3110 and MG1655ΔRM were used for EOP assays and for phage propagation (32). The EHEC O157:H7 strain EDL933 Δ*stx1/2* was used as host for the construction of phage defense mutants (58) and is referred to as EDL933 wild type (WT) throughout the manuscript, tables, and figures. For growth experiments, *E. coli* strains were cultivated at 37°C in LB under vigorous agitation, and the growth was monitored by measuring the optical density at 600 nm (OD_600_).

### Phage propagation, EOP, and phage infection assays in liquid medium

The 44 phages of the BASEL collection (32, 59) used in this work for the determination of the phage spectra and synergy of the three phage defense systems are listed in Table S7. The phages were propagated in 20 mL LB using the *E. coli* MG1655ΔRM strain lacking all known *E. coli* K-12 restriction systems (32), which was cultivated to an OD_600_ of 0.3 and inoculated with the phage at an MOI of 0.1. Upon phage propagation, the OD_600_ of the *E. coli* MG1655ΔRM culture decreased to 0.1, and 100 µL chloroform was added for 15 min, followed by centrifugation (5,000 rpm, 15 min, 4°C), and sterile filtration of the phage lysate. The phage titer was determined using the top agar assay on regular Petri dishes (9.4 cm). The LB top agar contained 0.5% agar and was freshly prepared. About 3 mL of top agar was inoculated with the *E. coli* strain MG1655ΔRM as a susceptible host strain, which was mixed with serial dilutions of the phage lysates, and poured as an overlay on solid LB agar plates. After overnight incubation of the LB top agar plates, PFUs were counted for each phage dilution, and the titer of the phage lysate was determined.

Quantitative EOP assays were applied to determine the phage spectra and synergistic interactions of the defense systems. For EOP assays, larger square Petri dishes (12 cm × 12 cm) were used, and 10 mL of top agar inoculated with 200 μL of an overnight culture of *E. coli* EDL933 Δ*stx1/2* WT or defense system mutant strains to overlay the solid LB agar plates. Afterward, serial dilutions of phage lysate stocks were prepared in sterile 0.9% NaCl solution, and 5 μL of each dilution was spotted onto the top agar plates and dried. The plates were incubated at 37°C for 24 h to determine PFUs in the serial dilutions. The EOP of a phage on the defense mutant strain was determined by calculating the ratio of PFUs of the defense mutant versus the EDL933 Δ*stx1/2* (WT) strain. EOP assays for each phage and mutant strain were performed in 3–4 biological replicate experiments. The statistics for the log_10_ EOP values were calculated for log_10_ PFU WT/defense mutants for each phage using Student’s unpaired one-tailed *t*-test of two samples of unequal variance (Welch’s *t*-test).

The synergy or additive protection of combined versus the single defense systems was calculated from the EOP results as an epistatic coefficient (ε) according to the formula: [log_10_EOP (WT/combined mutant)] – [log_10_EOP (WT/single mutant1)] – [log_10_EOP (WT/single mutant2)], which indicates the synergy score. Synergistic interaction is given when [log_10_EOP (WT/combined mutant)] > [log_10_EOP (WT/single mutant1)] + [log_10_EOP (WT/single mutant2)] + 1. Additive protection is given when max [log_10_ EOP (WT/single mutant1)], [log_10_ EOP (WT/single mutant2)] < [log_10_EOP (WT/combined mutant)] < [(log_10_EOP (WT/single mutant1)) + (log_10_EOP (WT/single mutant2))] + 1. The synergistic or additive interactions of the defense systems are shown as a green color code in Fig. 5, and the epistatic coefficients (ε) are presented in Table S4. The statistics for the synergy score were calculated using Student’s unpaired one-tailed *t*-test of two samples of unequal variance (Welch’s *t*-test) for the EOP comparisons of combined defense mutants (Δ*zor-dru*, Δ*dru-arm*, or Δ*zor-dru-arm*) versus the calculated additive EOP values of the two or three single system mutants (Δ*zorABE*, Δ*druHE*, Δarm*ABCD*).

For liquid phage infection experiments, the EHEC strains were grown in LB to an OD_600_ of 0.08 and infected with different MOIs of the phages to monitor the growth and collapse of the bacterial populations. The average growth curves from three biological replicates of non-infected controls and infected cultures were used for the calculation of the AUC for quantitative comparison of the additive or synergistic interactions of the defense systems. The formula ([OD1 + OD2]/2) × (t2 − t1), in which OD1 and OD2 are the OD_600_ values at time points t1 and t2, respectively, was used for the determination of the AUC for each time point of OD_600_ measurement along the growth, which was then summed up to yield the total AUC of each growth curve of phage-infected strains. The total AUC of the average growth curve of the phage-infected strain EDL933 Δ*stx1/2* (WT) and the single defense mutant strains was used for the calculation of the interactions of the two systems. Synergistic interactions of the defense systems were considered when the AUC of the WT strain was significantly greater than the calculated additive AUC of the single defense mutants. The statistics to reveal significantly higher AUC values of the WT versus the additive AUC of the single defense mutants were calculated using Student’s unpaired one-tailed *t*-test of two samples of unequal variance (Welch’s *t*-test).

### Construction of phage defense mutants and complemented strains in EHEC strain EDL933 Δ*stx1/2*

The single and combined defense system mutants of the Zorya II, Druantia III, and ARMADA II systems located on OI-172 were constructed as clean (scarless) deletions in the EHEC strain EDL933 Δ*stx1/2* by using the one-step inactivation method of Datsenko and Wanner (60). First, the chloramphenicol resistance (*cat*) cassette, which is flanked by FRT (FLP recognition target) sites, was amplified by PCR from the pKD3 plasmid using gene-specific primers (Table S8), containing ~50 bp homology arms corresponding to the sequences immediately up- and downstream of the target gene in the EDL933 Δ*stx1/2* genome. Next, the single or multiple defense system genes were replaced by the *cat* cassette via the pKD46-encoded λ red recombinase.

For mutant constructions, purified PCR products of the *cat* cassette (1 µg) were electroporated into electrocompetent *E. coli* EDL933 Δ*stx1/2* pKD46 cells using a 0.1 cm gap cuvette at 1.8 kV. Following electroporation, cells were recovered in 1 mL LB medium at 37 °C for 90 min and plated on LB agar supplemented with chloramphenicol (25 µg/mL). Plates were incubated overnight at 37°C. Putative mutants were screened by colony PCR using primers flanking the integration site (Table S8). The pKD46 plasmid was subsequently cured by incubation at 42°C, and scarless mutants were obtained via FLP-mediated excision of the *cat* cassette using the pCP20 plasmid, as previously described (60).

For complementation of the EDL933 Δ*stx1/2* Δ*druE*, Δ*armC*, and Δ*zorE* deletion mutants, the low-copy-number plasmid pWKS30 was used (61), and the *druE*, *armC*, and *zorE* genes were cloned into pWKS30 by Gibson assembly (62). The *armC* and *zorE* genes were amplified from the EDL933 Δ*stx1/2* genome. The vector pFL-*druE* was used as a template for the amplification of *druE*. For the construction of the pFL-*druE* vector, synthetic gene blocks encoding *druE*, codon-optimized for insect cell expression, were stepwise assembled and integrated into the pFL vector again using Gibson assembly (62). The pWKS30 plasmids encoding *druE*, *armC*, or *zorE* were electroporated into the respective defense mutants. The *druE*, *armC*, and *zorE* complemented strains were selected on LB agar plates containing ampicillin and analyzed in growth experiments with selected phages for restored phage defense after induction by 200 µM IPTG as compared to the respective mutants.

## Data Availability

The authors confirm that the data supporting the findings of this study are available within the article and its supplementary materials.

## SUPPLEMENTAL MATERIAL

Fig. S1 and S2 mbio.00943-26-s0001.pdf Growth curves and AUC comparison of defense mutants.

Tables S6 to S8 mbio.00943-26-s0002.pdf Bacterial strains, plasmids, phages, and oligonucleotide (primer) sequences.

Table S1 mbio.00943-26-s0003.xlsx Distribution of Zorya II, Druantia III, and ARMADA II based on the KEGG database.

Table S2 mbio.00943-26-s0004.xlsx Distribution of Zorya II, Druantia III, and ARMADA II in *E. coli* genomes from the NCBI RefSeq collection.

Table S3 mbio.00943-26-s0005.xlsx EHEC strain EDL933 is more resistant to phages compared to *E. coli* K-12 strain W3110.

Table S4 mbio.00943-26-s0006.xlsx Phage spectra and synergy of the Zorya II, Druantia III, and ARMADA II systems in EHEC strain EDL933.

Table S5 mbio.00943-26-s0007.xlsx Complementation of the helicase/nuclease effector mutants of the Zorya II, Druantia III, and ARMADA II defense systems restored their activity in the phage defense.

ASM does not own the copyrights to Supplemental Material that may be linked to, or accessed through, an article. The authors have granted ASM a non-exclusive, world-wide license to publish the Supplemental Material files. Please contact the corresponding author directly for reuse.
