# Supplementary material for: The co-localizing Zorya II, Druantia III, and ARMADA II defense systems on O-island 172 confer synergistic anti-phage defense in enterohemorrhagic Escherichia coli
Source: mBio. 2026 Jun 22;17(8):e00943-26. doi: 10.1128/mbio.00943-26 (PMC13463889; doi:10.1128/mbio.00943-26)
Supplement: Supplementary file 1 [file mbio.00943-26-s0001.pdf]

Figure S1

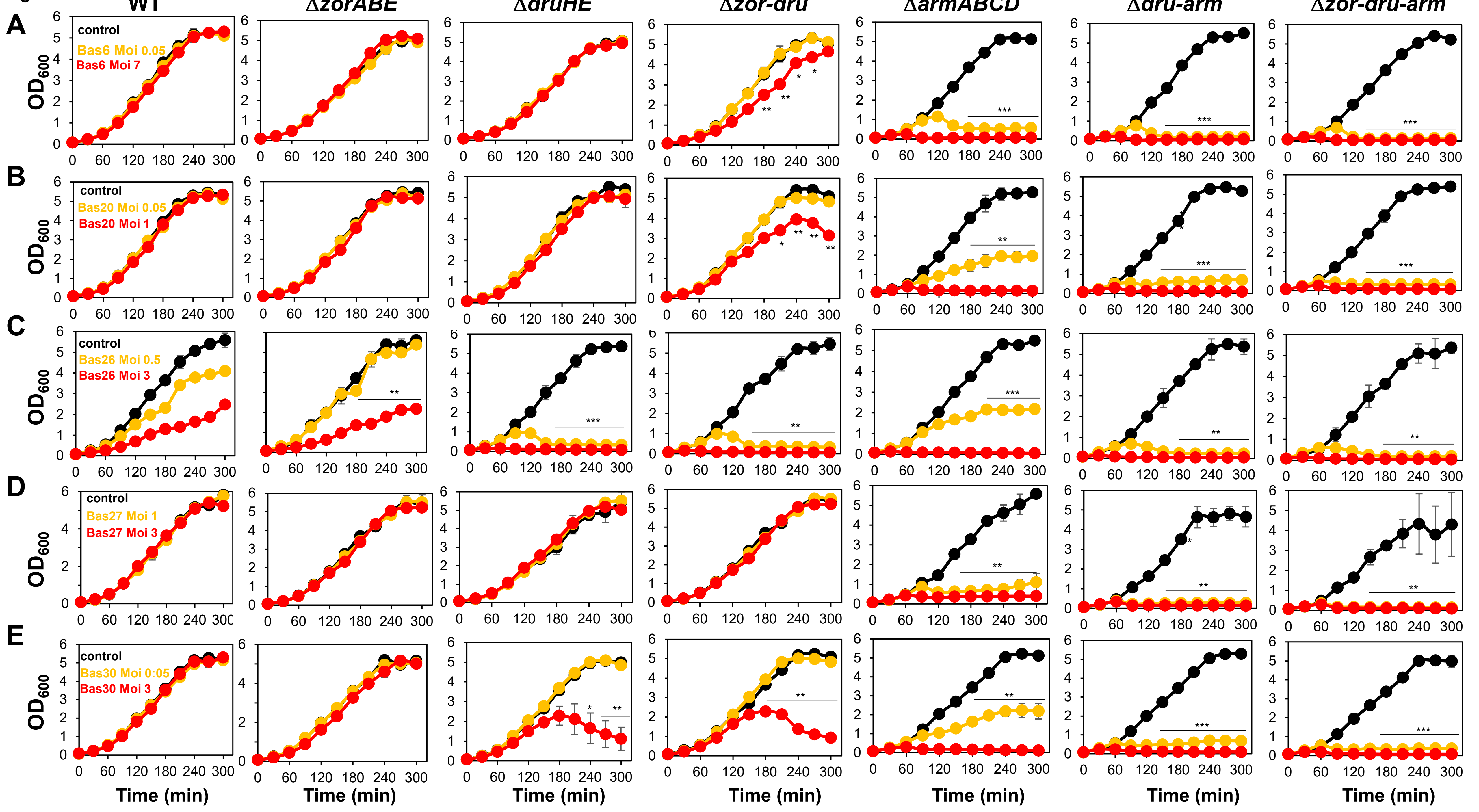
WT
ΔzorABE
ΔdruHE
Δzor-dru
ΔarmABCD
Δdru-arm
Δzor-dru-arm
A
B
C
D
E
OD_{600}
Time (min)
control
Bas6 Moi 0.05
Bas6 Moi 7
Bas20 Moi 0.05
Bas20 Moi 1
Bas26 Moi 0.5
Bas26 Moi 3
Bas27 Moi 1
Bas27 Moi 3
Bas30 Moi 0:05
Bas30 Moi 3

Figure S1 (continued)

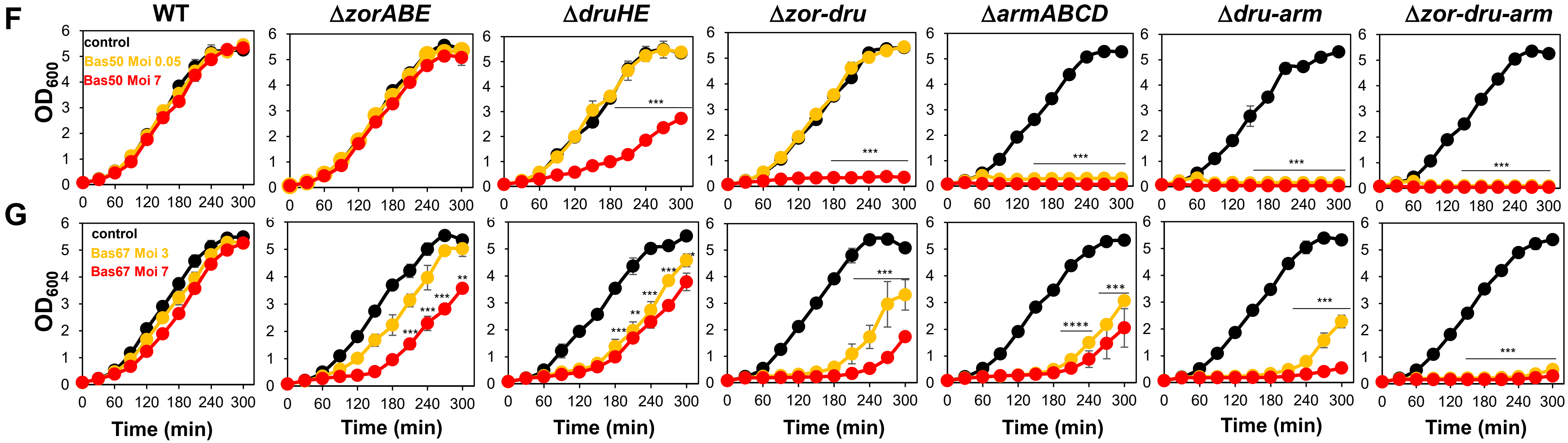

Fig. S1A-G. Growth curves of combined and single defense system mutants deficient in the Zorya II, Druantia III and/or ARMADA II systems. The growth curves of the EHEC strain EDL933 WT, *ΔzorABE, ΔdruHE* and *ΔarmABCD* single system and *Δzor-dru*, *Δdru-arm* and *Δzor-dru-arm* combined system mutants were monitored in LB medium after infection with Bas6, Bas20, Bas26, Bas27, Bas30, Bas 50 and Bas 67 at different MOIs. Average values of three biological replicates of the growth curves for the defense mutants are presented with error bars representing the SD. The statistics was calculated for the infected defense mutants versus the uninfected control using the Student's unpaired two-tailed t-test. *p*-values: *$p < 0.05$; **$p < 0.01$; ***$p < 0.001$; ****$p < 0.0001$. The growth curves were used for AUC calculation as described in the Methods section and presented in **Fig. S2**.

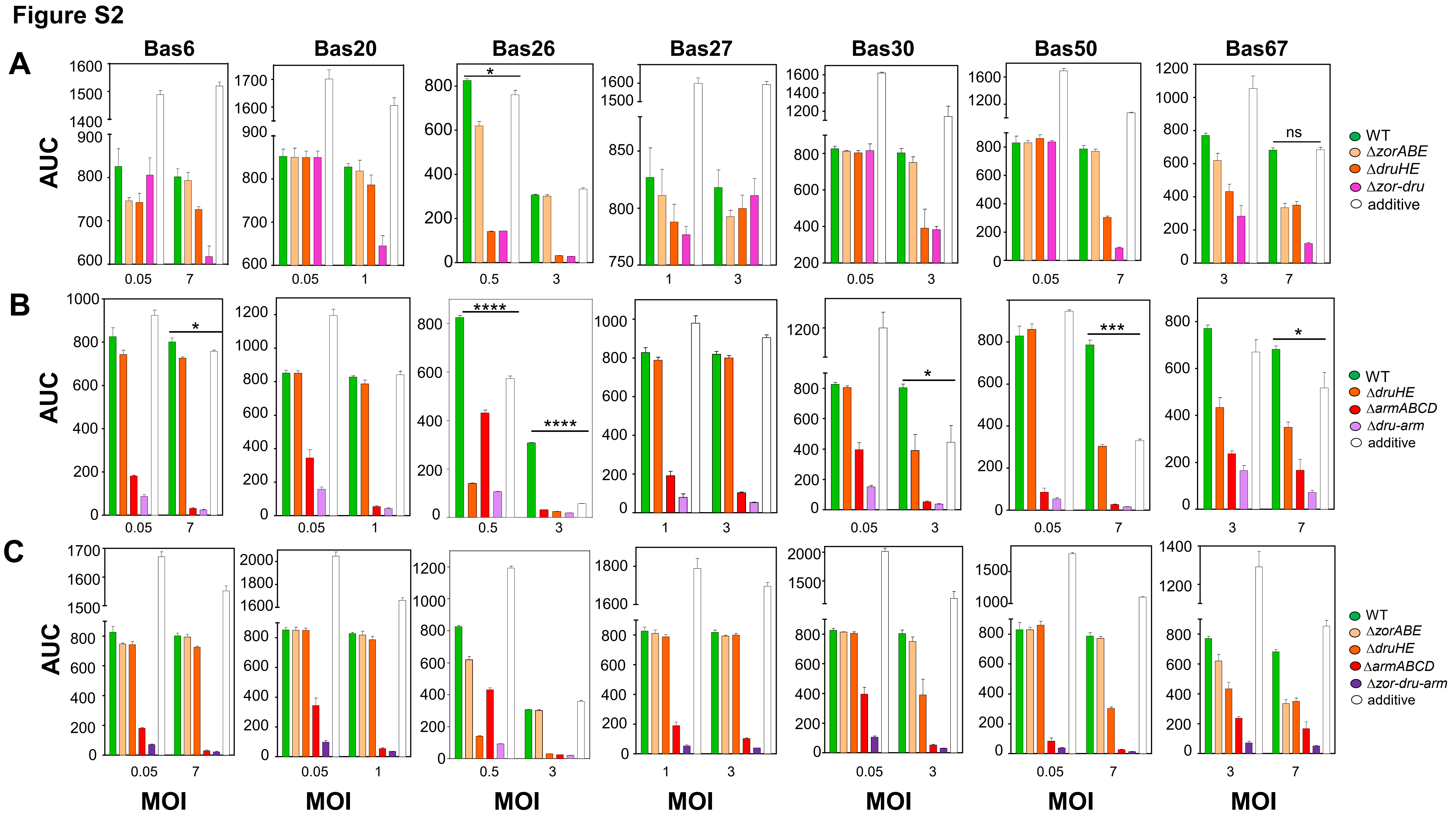
Figure S2
A
B
C
Bas6
Bas20
Bas26
Bas27
Bas30
Bas50
Bas67
AUC
MOI
WT
ΔzorABE
ΔdruHE
Δzor-dru
ΔarmABCD
Δdru-arm
Δzor-dru-arm
additive
ns
*
***
****

Figure S2 (continued)

D

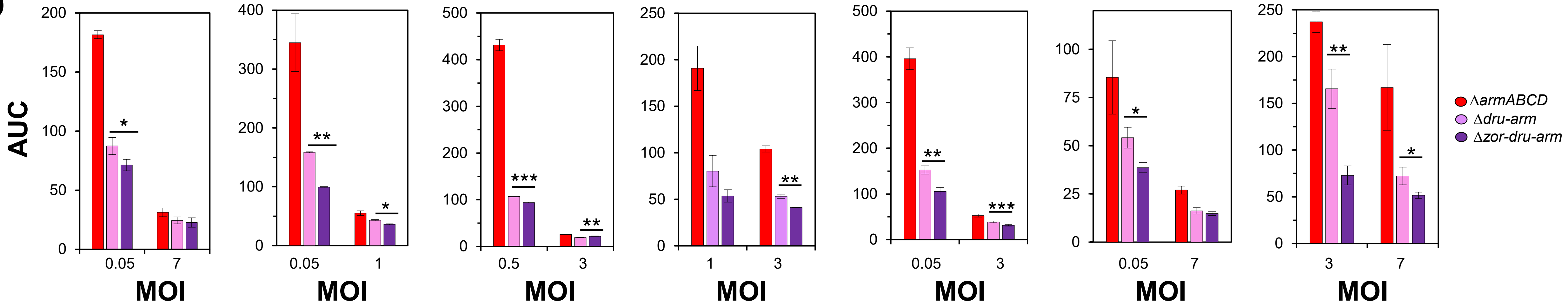

Fig. S2. Growth curves and AUC calculations support synergistic interactions of Druantia III and ARMADA II. Growth curves and AUC values were determined for *E. coli* EDL933 Δ*stx1/2* (WT) and the Δ*zorABE,* Δ*druHE*, and Δ*armABCD* single as well as Δ*zor-dru*, Δ*dru-arm*, and Δ*zor-dru-arm* combined system mutants in LB after infection with the phages Bas6, Bas20, Bas26, Bas27, Bas30, Bas 50, and Bas 67. The average growth curves from 3-4 biological replicates are presented in **Fig. S1A-G**. Interactions of Zorya II and Druantia III **(A)**, Druantia III and ARMADA II **(B)**, and Zorya II, Druantia III, and ARMADA II **(C)** were calculated as significant higher AUC values of the WT versus the additive AUC of the single system mutants using the Student's unpaired one-tailed *t*-test of two samples of unequal variance (Welch's *t*-test). For possible interaction of three defense systems **(D)**, significant differences of the Δ*dru-arm* versus the Δ*zor-dru-arm* mutants were calculated as above. *, $p < 0.05$; **, $p < 0.01$; ***, $p < 0.001$; ****, $p < 0.0001$.
