# Supplementary material for: The co-localizing Zorya II, Druantia III, and ARMADA II defense systems on O-island 172 confer synergistic anti-phage defense in enterohemorrhagic Escherichia coli
Source: mBio. 2026 Jun 22;17(8):e00943-26. doi: 10.1128/mbio.00943-26 (PMC13463889; doi:10.1128/mbio.00943-26)
Supplement: Supplementary file 2 [file mbio.00943-26-s0002.pdf]

Table S6. Bacterial strains and plasmids.

Strains	Description	Reference
Escherichia coli		
EDL933 Δ*stx1/2*	EDL933 *stx1/2* deletion mutant	(1)
EDL933 Δ*stx1/2* Δ*zorA*	EDL933 *stx1/2 zorA* (z5896) scar mutant	This study
EDL933 Δ*stx1/2* Δ*zorB*	EDL933 *stx1/2 zorB* (z5895) scar mutant	This study
EDL933 Δ*stx1/2* Δ*zorE*	EDL933 *stx1/2 zorE* (z5894) scar mutant	This study
EDL933 Δ*stx1/2* Δ*druH*	EDL933 *stx1/2 druH* (z5897) scar mutant	This study
EDL933 Δ*stx1/2* Δ*druE*	EDL933 *stx1/2 druE* (z5898) scar mutant	This study
EDL933 Δ*stx1/2* Δ*armA*	EDL933 *stx1/2 armA* (z5899) scar mutant	This study
EDL933 Δ*stx1/2* Δ*armB*	EDL933 *stx1/2 armB* (z5900) scar mutant	This study
EDL933 Δ*stx1/2* Δ*armC*	EDL933 *stx1/2 armC* (z5901) scar mutant	This study
EDL933 Δ*stx1/2* Δ*armD*	EDL933 *stx1/2 armD* (z5902) scar mutant	This study
EDL933 Δ*stx1/2* Δ*zorABE*	EDL933 *stx1/2 zorABE* (z5896-94) scar mutant	This study
EDL933 Δ*stx1/2* Δ*druHE*	EDL933 *stx1/2 druHE* (z5897-98) scar mutant	This study
EDL933 Δ*stx1/2* Δ*armABCD*	EDL933 *stx1/2 armABCD* (z5899-5902) scar mutant	This study
EDL933 Δ*stx1/2* Δ*zorABE* Δ*druHE*	EDL933 *stx1/2 zorABE druHE* (z5894-5898) scar mutant	This study
EDL933 Δ*stx1/2* Δ*druHE* Δ*armABCD*	EDL933 *stx1/2 druHE armABCD* (z5897-5902) scar mutant	This study
EDL933 Δ*stx1/2* Δ*zorABE* Δ*druHE* Δ*armABCD*	EDL933 *stx1/2 zorABE druHE armABCD* (z5894-5902) scar mutant	This study
EDL933 Δ*stx1/2* Δ*druE*-pWKS30	EDL933 *stx1/2 druE* (z5898) scar mutant complemented with pWKS30 (empty plasmid)	This study
EDL933 Δ*stx1/2* Δ*druE*-pWKS30-*druE*	EDL933 *stx1/2 druE* (z5898) scar mutant complemented with pWKS30-*druE*	This study
EDL933 Δ*stx1/2* Δ*armC*-pWKS30	EDL933 *stx1/2 armC* (z5901) scar mutant complemented with pWKS30 (empty plasmid)	This study
EDL933 Δ*stx1/2* Δ*armC*-pWKS30-*armC*	EDL933 *stx1/2 armC* (z5901) scar mutant complemented with pWKS30-*armC*	This study
EDL933 Δ*stx1/2* Δ*zorE* pWKS30	EDL933 *stx1/2 zorE* (z5894) scar mutant complemented with pWKS30 (empty plasmid)	This study
EDL933 Δ*stx1/2* Δ*zorE* pWKS30-*zorE*	EDL933 *stx1/2 zorE* (z5894) scar mutant complemented with pWKS30-*zorE*	This study
DH5α	F-φ80dlacZ Δ(lacZYA-argF) U169 deoRsupE44ΔlacU169 (f80lacZDM15) hsdR17 recA1 endA1 (rk- mk+) supE44gyrA96 thi-1 gyrA69 relA1	(2)
K-12 MG1655 ΔRM	K-12 MG1655 ΔRM	(3)
W3110	F^- $lambda^-$ IN(rrnD-rrnE)1 rph-1	(4)
**Plasmids**		
pKD46	Amp^R, λ Red recombinase expression	(5)
pKD3	Amp^R, Cm cassette template	(5)
pCP20	*FLP*$^+$, λcI857+, λP_R Rep^{ts}, Amp^R, Cm^R	(6)
pWKS30	Amp^R, *lacZ*α, pSC101 *ori*, f1 *ori*, MCS	(7)
pWKS30-*druE*	pWKS30 expressing *druE* under *lac*-promoter	This study
pWKS30-*armC*	pWKS30 expressing *armC* under *lac*-promoter	This study
pWKS30-*zorE*	pWKS30 expressing *zorE* under *lac*-promoter	This study
pFL	Amp^R, Gen^R. f1 *ori*, MCS, polyhedron and p10 promotor, 10His, TEV site	(8)
pFL-*druE*	pFL expressing *druE* under the polyhedron promotor	This study

^{R}: resistant, Amp: ampicillin, Cm: chloramphenicol, Gen: gentamicin

Table S7. Phages used in this study

Phage number	ICTV Phage taxonomy https://ictv.global/taxonomy	Reference/Source
Bas3	Class: *Caudoviricetes*, Family: *Drexlerviridae*	(3, 9)
Bas5	Class: *Caudoviricetes*, Family: *Drexlerviridae*	(3, 9)
Bas6	Class: *Caudoviricetes*, Family: *Drexlerviridae*	(3, 9)
Bas7	Class: *Caudoviricetes*, Family: *Drexlerviridae*	(3, 9)
Bas13	Class: *Caudoviricetes*, Family: *Drexlerviridae*	(3, 9)
Bas18	Class: *Caudoviricetes*, Genus: *Dhillonvirus*	(3, 9)
Bas19	Class: *Caudoviricetes*, Subfamily: *Queuovirinae*	(3, 9)
Bas20	Class: *Caudoviricetes*, Subfamily: *Queuovirinae*	(3, 9)
Bas22	Class: *Caudoviricetes*, Subfamily: *Queuovirinae*	(3, 9)
Bas23	Class: *Caudoviricetes*, Subfamily: *Queuovirinae*	(3, 9)
Bas26	Class: *Caudoviricetes*, Family: *Demerecviridae*	(3, 9)
Bas27	Class: *Caudoviricetes*, Family: *Demerecviridae*	(3, 9)
Bas28	Class: *Caudoviricetes*, Family: *Demerecviridae*	(3, 9)
Bas29	Class: *Caudoviricetes*, Family: *Demerecviridae*	(3, 9)
Bas30	Class: *Caudoviricetes*, Family: *Demerecviridae*	(3, 9)
Bas32	Class: *Caudoviricetes*, Family: *Demerecviridae*	(3, 9)
Bas33	Class: *Caudoviricetes*, Family: *Demerecviridae*	(3, 9)
Bas34	Class: *Caudoviricetes*, Family: *Demerecviridae*	(3, 9)
T5	Class: *Caudoviricetes*, Family: *Demerecviridae*	(3, 9)
T4	Class: *Caudoviricetes*, Family: *Straboviridae*	(3, 9)
T6	Class: *Caudoviricetes*, Family: *Straboviridae*	(3, 9)
Bas36	Class: *Caudoviricetes*, Family: *Straboviridae*	(3, 9)
Bas44	Class: *Caudoviricetes*, Family: *Straboviridae*	(3, 9)
Bas45	Class: *Caudoviricetes*, Family: *Straboviridae*	(3, 9)
Bas46	Class: *Caudoviricetes*, Family: *Straboviridae*	(3, 9)
#41	Class: *Caudoviricetes*, Family: *Straboviridae*	Thomas Gaudin
Bas49	Class: *Caudoviricetes*, Subfamily: *Vequintavirinae*	(3, 9)
Bas50	Class: *Caudoviricetes*, Subfamily:*Vequintavirinae*	(3, 9)
Bas51	Class: *Caudoviricetes*, Subfamily:*Vequintavirinae*	(3, 9)
Bas54	Class: *Caudoviricetes*, Subfamily: *Vequintavirinae*	(3, 9)
Bas55	Class: *Caudoviricetes*, Subfamily: *Vequintavirinae*	(3, 9)
Bas56	Class: *Caudoviricetes*, Subfamily: *Vequintavirinae*	(3, 9)
Bas57	Class: *Caudoviricetes*, Subfamily: *Vequintavirinae*	(3, 9)
Bas60	Class: *Caudoviricetes*, Subfamily: *Stephanstirmvirinae*	(3, 9)
Bas61	Class: *Caudoviricetes*, Subfamily: *Stephanstirmvirinae*	(3, 9)
Bas62	Class: *Caudoviricetes*, Subfamily: *Stephanstirmvirinae*	(3, 9)
Bas64	Class: *Caudoviricetes*, Order: *Autographivirales*	(3, 9)
Bas65	Class: *Caudoviricetes*, Order: *Autographivirales*	(3, 9)
T7	Class: *Caudoviricetes*, Order: *Autographivirales*	(3, 9)
Bas66	Class: *Caudoviricetes*, Order: *Autographivirales*	(3, 9)
Bas67	Class: *Caudoviricetes*, Order: *Autographivirales*	(3, 9)
Bas68	Class: *Caudoviricetes*, Order: *Autographivirales*	(3, 9)
λ *vir*	Class: *Caudoviricetes*, Genus: *Lambdavirus*	(3, 9)
phi80	Class: *Caudoviricetes*, Genus: *Enterobacteria phage phi80*	Lab stock

Table S8. Oligonucleotide (primer) sequences

Oligo name	Sequence
**Primers for amplifying the pWKS30 backbone**	
pWKS30_rev	AGCTGTTTCCTGTGTGAAATTGTT
pWKS30_fwd	ACCATGATTACGCCAAGCGC
**Primers for amplifying *druE* from pFL-*druE***	
*druE*_fwd	CAATTTCACACAGGAAACAGCTATGGACGTTCAGCAGCTGC
*druE*_rev	ATTGCGCGCTTGGCGTAATCATGGTTTATCCGGCAATGATGTAAGACC
**Primers for amplifying genes from the EDL933 Δ*stx1/2* genome**	
*armC*_fwd	CACACAGGAAACAGCTATGTTGCCGTCCGTTGTCAG
*armC*_rev	CGCGCTTGGCGTAATCATGGTTTATTTGTCACCTCCGGACAG
*zorE*_fwd	CAATTTCACACAGGAAACAGCTATGAAATTATCTATCGACATTTCAGAAC
*zorE*_rev	CGCTTGGCGTAATCATGGTTTACAATTTTGCTGGCGTAAAG
**Primers for cloning gene block 1 into pFL vector**	
pFL_EcoRI_fw	GGTTCAGCAGAGGGGCGTAGTGCAGCTGCTGAACGTCCATGAATTCGGCGCCC TGAAAATAAAGATTC
pFL_block1_rv	CTATCGACGCCCTGAAGAAAGCTATGGCCGCCAACGAAGAACAAGCTTGTCGA GAAGTACTAGAGGATC
block1_fw	TTCCATGGACGTTCAGCAGCTGCAC
block1_rv	GTTCTTCGTTGGCGGCCATAGCTTTC
**Primers for cloning gene block 2 and 3 into block 1-containing pFL vector**	
block2_fw	GTCCTGCCTCTGAAGAACTCTATCGACGC
block3_rv	TTATCCGGCAATGATGTAAGACCTC
pFL-block1_rv	GTTCTTCGTTGGCGGCCATAGCTTTC
pFL-block1_fw	CTCCTGGAAACGACCTGAGGTCTTACATCATTGCCGGATAAAAGCTTGTCGAGA AGTACTAGAGGATC
**Primers for construction of *scar* defense deletion mutants in EDL933 Δ*stx1/2***	
mutZ5894-cm-for	GTAACAAATGCCGAGTTGCAGATTAGAAAGATTATTCAGGAGTAAGAGATGAGT GTAGGCTGGAGCTGCTTC
mutZ5894-cm-rev	TTATTTATCTTCCGTGAAAGTCTTAACTTTCACGGAGTTTTAGAATTTACATATGA ATATCCTCCTTAGTTCC
Control-5894-for	GTAACAAATGCCGAGTTGCAGA
Control-5894-rev	CTTCCGTGAAAGTCTTAACTTTC
mutZ5895-cm-for	ATGGATAAGATTATAGGGAAACAATTACCTAAAAAAGATCAAGATAATGGTGTAG GCTGGAGCTGCTTC
mutZ5895-cm-rev	TTACTCCTGAATAATCTTTCTAATCTGCAACTCGGCATTTGTTACCATATGAATAT CCTCCTTAGTTCC
Control-5895-for	ATGGATAAGATTATAGGGAAAC
Control-5895-rev	CTCCTGAATAATCTTTCTAATC
mutZ5896-cm-for	TAAACGATTACAACTATCCATTATTTACACAGGTATCAAAATGTTAGCGCGTGTA GGCTGGAGCTGCTTC
mutZ5896-cm-rev	TTACCCGCGATGTTTATTAGCAGAAATTAATGCTTGTAACTGCCCATATGAATATC CTCCTTAGTTCC
Control-5896-for	CGATTACAACTATCCATTATTTAC
Control-5896-rev	CGCGATGTTTATTAGCAGAAAT
mutZ5897-cm-for	GTCGCCGTTCATTATCCGGAGCTGATGGACGGGCGTTATATGCTTACCGTGTGT AGGCTGGAGCTGCTTC
mutZ5897-cm-rev	ATTGCTGTGGGGCTTGCTGATAGTGAAATTCAAAAAGCGCAGACAGTTGACATA TGAATATCCTCCTTAGTTCC
Control-5897-for	CGTTCATTATCCGGAGCTGATG

Control-5897-rev	GTGAAATTCAAAAAGCGCAGACAG
mutZ5898-cm-for	GGGGTAATCTATCAATATTATTTTGAACAAGCAGGAGTATGTCATTGATGGTGTA GGCTGGAGCTGCTTC
mutZ5898-cm-rev	ATCCCCAGGGGAGCAATTATTTATCCAGCGATAATATAACTCCTAAGGTCTGAAT ATCCTCCTTAGTTCC
mutZ5899-cm-for	ATGAATAAGCAAAATTATGCACCTGGTATGCGGGTGGTTATTCGTGATGCGTGTA GGCTGGAGCTGCTTC
mutZ5899-cm-rev	TTAACCCTCCGCTCCGGTGATAACAGCAATTACCTGAATGTAGGGCATATGAATA TCCTCCTTAGTTCC
Control-5899-for	ATGAATAAGCAAAATTATGCACC
Control-5899-rev	CCGGTGATAACAGCAATTAC
mutZ5900-cm-for	ATGGCGCTGGTCGGTATTAATAACGAAAACGAATTTTACTCTAACCACTAGTGTA GGCTGGAGCTGCTTC
mutZ5900-cm-rev	CAGGCGCTCTCCTTATCTTGTGCAAAGAACGCCCAGGCCACTTTGCATATGAAT ATCCTCCTTAGTTCC
Control-5900-for	CGCTGGTCGGTATTAATAAC
Control-5900-rev	CGCTCTCCTTATCTTGTGCA
mutZ5901-cm-for	CTACAAAGTGGCCTGGGCGTTCTTTGCACAAGATAAGGAGAGCGCCTGATGTTG GTGTAGGCTGGAGCTGCTTC
mutZ5901-cm-rev	GCCCATTTTTGTGATGGCCAGGCGAGATCGGCTTCCGCAATAATTTCACCTTCC ATATGAATATCCTCCTTAGTTCC
Control-5901-for	CTACAAAGTGGCCTGGGCGT
Control-5901-rev	GCCCATTTTTGTGATGGCCA
mutZ5902-cm-for	CGGTGCAAACGCCCAAGGTGGCACTTTCTGATGGTTTTCTGGGCGCATTCGTGT AGGCTGGAGCTGCTTC
mutZ5902-cm-rev	TCAGTCAGCCACTAATAGCGGACTAGGGACACCAGATGATGAAACCATATGAAT ATCCTCCTTAGTTCC
Control-5902-for	GCAAACGCCCAAGGTGGCA
Control-5902-rev	TCAGTCAGCCACTAATAGCG
mutZ5899-5902-cm-for	CGAACAGCAACTGCGAGCGGATCTGAAATTGAAAGATGACCAGATCGCCAGTGT AGGCTGGAGCTGCTTC
mutZ5899-5902-cm-rev	ATCGCTGTCGTATACCACGCGGAAATCATAACCGTGGCGTTTTAGCATATGAATA TCCTCCTTAGTTCC
mutZ5894-5898-cm-for	CTTCCGTGAAAGTCTTAACTTTCACGGAGTTTTAGAATTTACAATTTTGGTGTAGG CTGGAGCTGCTTC
mutZ5894-5898-cm-rev	ATCCCCAGGGGAGCAATTATTTATCCAGCGATAATATAACTCCTAAGGTCCATAT GAATATCCTCCTTAGTTCC
Control-mutZ5894-5898- for	CTACTTTTGTAATTTGAGCCAC
Control-mut5894-5898-rev	GCATAAATTGCGCATGATTTTC
mutZ5897-5902-cm-for	ATGATAGTAACCTGTTACGAATTGTATCAAAGGAAGGCAGGTTGCTTGTGGTGTA GGCTGGAGCTGCTTC

Restriction sites are underlined and bold bases indicate point mutations

Supplementary References

1. Gobert AP, Vareille M, Glasser AL, Hindre T, de Sablet T, Martin C. 2007. Shiga toxin produced by enterohemorrhagic *Escherichia coli* inhibits PI3K/NF-kappaB signaling pathway in globotriaosylceramide-3-negative human intestinal epithelial cells. J Immunol 178:8168-74.

2. Studier FW, Moffatt BA. 1986. Use of bacteriophage T7 RNA polymerase to direct selective high-level expression of cloned genes. J Mol Biol 189:113-30.

3. Maffei E, Shaidullina A, Burkolter M, Heyer Y, Estermann F, Druelle V, Sauer P, Willi L, Michaelis S, Hilbi H, Thaler DS, Harms A. 2021. Systematic exploration of *Escherichia coli* phage-host interactions with the BASEL phage collection. PLoS Biol 19:e3001424.

4. Hayashi K, Morooka N, Yamamoto Y, Fujita K, Isono K, Choi S, Ohtsubo E, Baba T, Wanner BL, Mori H, Horiuchi T. 2006. Highly accurate genome sequences of *Escherichia coli* K-12 strains MG1655 and W3110. Mol Syst Biol 2:2006 0007.

5. Datsenko KA, Wanner BL. 2000. One-step inactivation of chromosomal genes in *Escherichia coli* K-12 using PCR products. Proc Natl Acad Sci U S A 97:6640-5.

6. Cherepanov PP, Wackernagel W. 1995. Gene disruption in *Escherichia coli*: TcR and KmR cassettes with the option of Flp-catalyzed excision of the antibiotic-resistance determinant. Gene 158:9-14.

7. Wang RF, Kushner SR. 1991. Construction of versatile low-copy-number vectors for cloning, sequencing and gene expression in *Escherichia coli*. Gene 100:195-9.

8. Fitzgerald DJ, Berger P, Schaffitzel C, Yamada K, Richmond TJ, Berger I. 2006. Protein complex expression by using multigene baculoviral vectors. Nat Methods 3:1021-32.

9. Humolli D, Piel D, Maffei E, Heyer Y, Agustoni E, Shaidullina A, Willi L, Imwinkelried P, Estermann F, Cuenod A, Buser DP, Alampi C, Chami M, Egli A, Hiller S, Dunne M, Harms A. 2025. Completing the BASEL phage collection to unlock hidden diversity for systematic exploration of phage-host interactions. PLoS Biol 23:e3003063.
